# Systematic mutagenesis of TFIIH subunit p52/Tfb2 identifies residues required for XPB/Ssl2 subunit function and genetic interactions with *TFB6*

**DOI:** 10.1016/j.jbc.2022.102433

**Published:** 2022-08-28

**Authors:** Jacob Bassett, Jenna K. Rimel, Shrabani Basu, Pratik Basnet, Jie Luo, Krysta L. Engel, Michael Nagel, Alexander Woyciehowsky, Christopher C. Ebmeier, Craig D. Kaplan, Dylan J. Taatjes, Jeffrey A. Ranish

**Affiliations:** 1 Department of Systems Biology, Institute for Systems Biology, Seattle, Washington, USA; 2 Department of Biochemistry, University of Colorado, Boulder, Colorado, USA; 3 Department of Cell Biology, University of Pittsburgh, Pennsylvania, USA; 4 Department of Biological Sciences, University of Pittsburgh, Pittsburgh, Pennsylvania, USA; 5 Boulder BioConsulting, Inc, Boulder, Colorado, USA

**Keywords:** general transcription factor TFIIH, p52/Tfb2, XPB/Ssl2, GTF2H4, Tfb6, RNA polymerase II, structure, function, mutagenesis, transcription start site, AGC, automatic gain control, CAK, CDK-activating kinase, ChIP, chromatin immunoprecipitation, CSM Leu−, complete synthetic medium minus leucine, EB, extraction buffer, 5-FOA, 5-fluoroorotic acid, HEK293T, human embryonic kidney 293T cell line, IgG, immunoglobulin G, IP, immunoprecipitation, MPA, mycophenolic acid, MS, mass spectrometry, NTD, N-terminal domain, pA, protein A, PIC, preinitiation complex, pol II, polymerase II, TEV, tobacco etch virus, TSS, transcription start site, WCE, whole-cell extract

## Abstract

TFIIH is an evolutionarily conserved complex that plays central roles in both RNA polymerase II (pol II) transcription and DNA repair. As an integral component of the pol II preinitiation complex, TFIIH regulates pol II enzyme activity in numerous ways. The TFIIH subunit XPB/Ssl2 is an ATP-dependent DNA translocase that stimulates promoter opening prior to transcription initiation. Crosslinking-mass spectrometry and cryo-EM results have shown a conserved interaction network involving XPB/Ssl2 and the C-terminal Hub region of the TFIIH p52/Tfb2 subunit, but the functional significance of specific residues is unclear. Here, we systematically mutagenized the HubA region of Tfb2 and screened for growth phenotypes in a *TFB6* deletion background in *Saccharomyces cerevisiae*. We identified six lethal and 12 conditional mutants. Slow growth phenotypes of all but three conditional mutants were relieved in the presence of *TFB6*, thus identifying a functional interaction between Tfb2 HubA mutants and Tfb6, a protein that dissociates Ssl2 from TFIIH. Our biochemical analysis of Tfb2 mutants with severe growth phenotypes revealed defects in Ssl2 association, with similar results in human cells. Further characterization of these *tfb2* mutant cells revealed defects in *GAL* gene induction, and reduced occupancy of TFIIH and pol II at *GAL* gene promoters, suggesting that functionally competent TFIIH is required for proper pol II recruitment to preinitiation complexes *in vivo*. Consistent with recent structural models of TFIIH, our results identify key residues in the p52/Tfb2 HubA domain that are required for stable incorporation of XPB/Ssl2 into TFIIH and for pol II transcription.

The evolutionarily conserved general transcription and DNA repair factor TFIIH plays central roles in both RNA polymerase II (pol II) transcription and in nucleotide excision repair. The 10-subunit complex consists of a seven-subunit core complex and a dissociable three-subunit CDK-activating kinase (CAK) module. The core consists of two ATPase-containing subunits XPB (human)/Ssl2 (yeast) and XPD/Rad3 as well as p62/Tfb1, p52/Tfb2, p44/Ssl1, p34/Tfb4, and p8/Tfb5, whereas the CAK module, known as TFIIK in yeast, consists of Cdk7/Kin28, CcnH/CycH, and Mat1/Tfb3 (human/yeast). During pol II transcription, TFIIH uses the ATP-dependent double-stranded DNA translocase activity of XPB/Ssl2 to stimulate promoter opening (1, 2, 3, 4, 5), and it uses the kinase activity of Cdk7/Kin28 to phosphorylate the C-terminal domain of the pol II Rpb1 subunit that occurs during the transition from initiation to elongation (6, 7, 8). The enzymatic activity of XPD/Rad3 is not required for transcription (1, 9). During nucleotide excision repair, the CAK is replaced by XPA, the ATPase activity of XPB/Ssl2 is needed to anchor TFIIH to sites of damage, and the helicase activity of XPD/Rad3 is used to scan for the lesion (10, 11, 12).

Until recently, our understanding of the molecular architecture of TFIIH, and how its enzymatic activities are regulated, has been limited (4, 13, 14, 15, 16). Prior to the publication of the high-resolution cryo-EM structures of human and yeast TFIIH (17, 18), we determined the molecular architecture of native human and yeast TFIIH complexes and found an interaction network involving the N- and C-terminal regions of XPB, the C-terminal Hub region of p52/Tfb2, and p8/Tfb5 (19). The Hub region of p52/Tfb2 consists of the N-terminal HubA (also known as the Clutch domain (18) amino acids 306–400/338–450, p52/Tfb2), and the C-terminal p8/Tfb5 dimerization domain (20) (amino acids 400–462/450–513, p52/Tfb2). The HubA region is conserved between human and yeast (47% identity/67% similarity). The cryo-EM structures of TFIIH confirmed the importance of p52/Tfb2 and p8/Tfb5 for anchoring XPB/Ssl2 to core TFIIH. The structures show that the N-terminal domain (NTD) of XPB/Ssl2 interacts with the HubA region of p52/Tfb2 by forming a pseudosymmetric dimer of structurally homologous domains, and the second RecA domain of XPB/Ssl2 contacts p8/Tfb5 (17, 18). Furthermore, both p52/Tfb2 and p8/Tfb5 can stimulate the ATPase activity of XPB (14, 15) in an additive fashion (21) indicating an important role for both subunits in regulating the translocase activity of XPB and promoter opening (22).

To identify residues in the p52/Tfb2 HubA region that are important for the function of TFIIH, we systematically mutagenized this region in *TFB2* and screened for growth phenotypes. We identified six lethal and 12 conditional mutants in a *tfb6*Δ background. *TFB6* is a nonessential gene whose protein product dissociates Ssl2 from TFIIH (23). No ortholog of Tfb6 is encoded in the human genome, although Tfb6 displays sequence similarity to human nuclear RNA export factor 5. Mapping the mutations onto the recent cryo-EM structures of TFIIH revealed that they clustered into distinct regions, allowing three groups to be distinguished: group 1 mutations map to the predicted interface between HubA and the XPB/Ssl2 Clutch domain; group 2 mutations map to the internal region of HubA; and group 3 mutations map to the C-terminal region of the HubA domain. Interestingly, the slow growth phenotypes of all group 2 mutants were relieved in a *TFB6* background indicating a synthetic interaction between group 2 *tfb2* alleles and *tfb6Δ*. Biochemical analysis of human and yeast group 1 p52/Tfb2 mutants revealed that the mutated residues are required for stable incorporation of XPB/Ssl2 into TFIIH, and functional analysis in yeast cells expressing these mutants revealed defects in preinitiation complex (PIC) formation and transcription. Surprisingly, we also found that Tfb6 is required for normal levels of TFIIH and PIC assembly in cells expressing group 2 mutant subunits. The results point to a feedback loop involving Tfb6 and TFIIH subunits designed to maintain appropriate levels of holo-TFIIH.

## Results

### Systematic mutagenesis of the p52/Tfb2 HubA region

**Figure 1:**
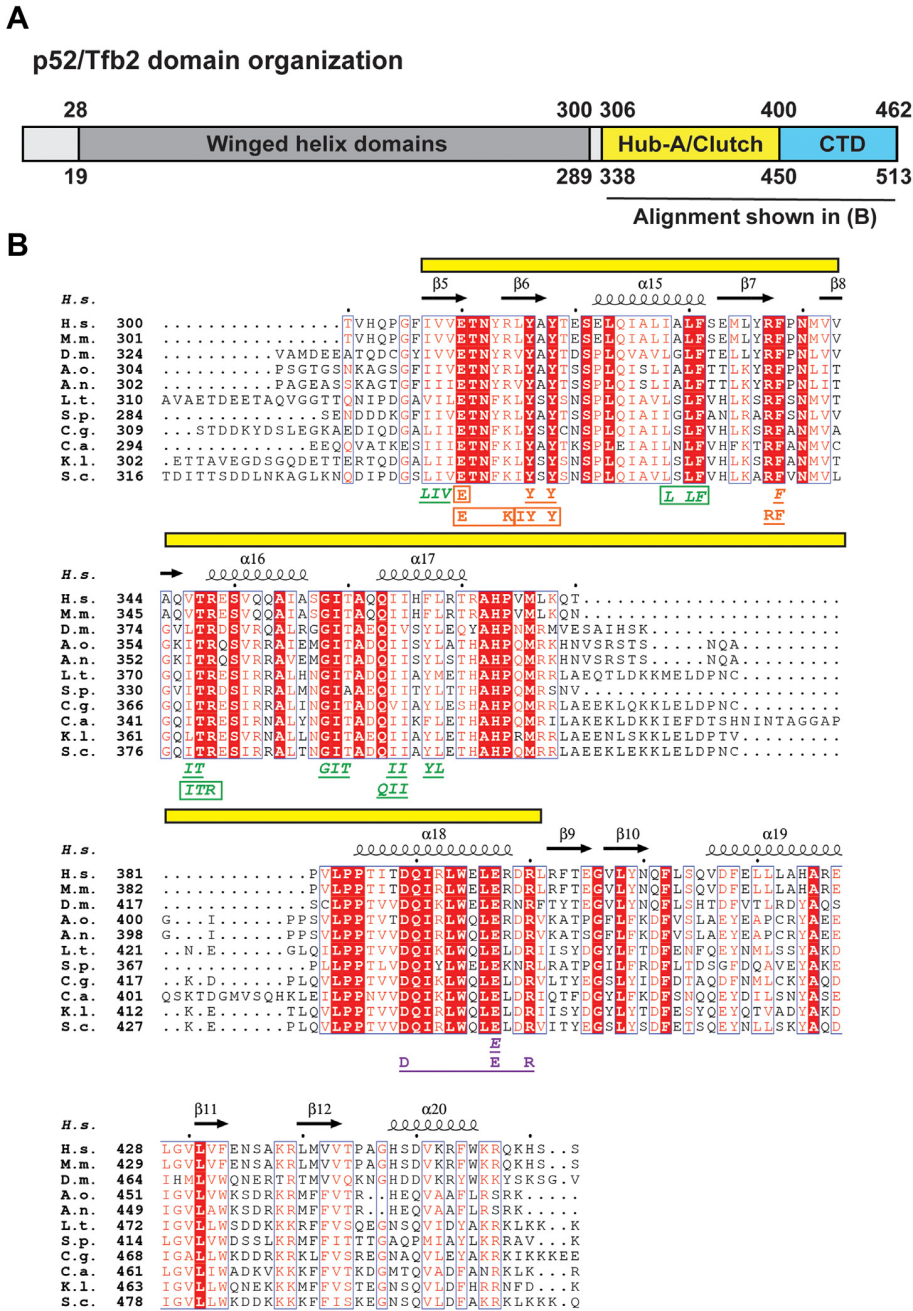
**Domain organization of p52/Tfb2, sequence alignment of the HubA domain of p52/Tfb2 from eukaryotes, and summary of mutants found in this study.***A*, schematic of the domain organization of human p52 (*top*) and *Saccharomyces cerevisiae* Tfb2 (*bottom*). *B*, multiple sequence alignment of the C terminus of p52/Tfb2 and summary of mutants. Identical residues are printed *white* on *red* background, and similar residues are printed in *red* on *white* background. The *yellow bar* indicates the HubA region mutagenized in this study. Secondary structure elements are indicated. Group 1, 2, and 3 mutants (*orange*, *green*, and *magenta*, respectively) found in this study are indicated below the alignment. Lethal mutants are *boxed*, mutants that display a slow growth phenotype in the absence of *TFB6* are in *italics*, and mutants that require *TFB6* for viability are in *italics* and *boxed*. Figure was generated using the ESPript web server (52). A.n., *Aspergillus niger*; A.o., *Aspergillus oryzae*; C.a., *Candida albicans*; C.g., *Candida glabrata*; D.m., *Drosophila melanogaster*; H.s., *Homo sapiens*; K.l., *Kluyveromyces lactis*; L.t., *Lachancea thermotolerans*; M.m., *Mus musculus*; S.c., *Saccharomyces cerevisiae*; S.p., *Schizosaccharomyces pombe.*

We previously determined the molecular architecture of native human and yeast TFIIH and found an interaction network involving the C-terminal Hub region of p52/Tfb2, p8/Tfb5, and the N- and C-terminal lock regions of XPB/Ssl2 (19). Recent high-resolution structures of human and yeast TFIIH confirm this interaction network (5, 17). The Hub region of p52/Tfb2 consists of an N-terminal HubA domain (18) (amino acids 306–400/338–450, p52/Tfb2), which was recently shown to assume the same overall fold as the NTD of XPB (5, 17), and the C-terminal p8/Tfb5 dimerization domain (amino acids 400–462/450–513 p52/Tfb2) (20) (Fig. 1*A*). The Hub region contains one of the most conserved stretches of amino acids in p52/Tfb2 (16) (Fig. 1*B*).

To further localize functionally important regions of HubA, we generated a series of small internal deletions throughout HubA in *Saccharomyces cerevisiae* Tfb2, from amino acids 339 to 410, and evaluated the ability of the mutants to support cell growth (Fig. S1). The phenotypes of the strains expressing the mutants were analyzed by yeast plasmid shuffle assay, where the mutant copy of *TFB2* was substituted for the WT copy. None of the mutants supported growth. These results are consistent with the recent finding that HubA forms a compact folded structure (5, 17) that would be disrupted by small internal deletions.

To identify residues in HubA that are important for the function of p52/Tfb2, we systematically mutagenized the region in Tfb2 using alanine substitutions and radical mutations (Tables 1 and S1) and evaluated the ability of the mutants to support cell growth at 20, 30, and 37 °C using the plasmid shuffle strategy (Fig. S2). We focused on residues that are conserved between the human and yeast homologs. To stabilize Ssl2 during immunoprecipitation (IP) analysis of the Tfb2 variants, the plasmid shuffle strains used in the initial screens contained a deletion of *TFB6*, a nonessential gene that facilitates dissociation of Ssl2 from TFIIH (23). In our first round of mutagenesis, we changed 46 residues from amino acids 342 to 450 to alanine. None of the alanine substitution mutants yielded a growth phenotype except for F370A, which had a slight slow growth phenotype at 37 °C.

We next created a set of 21 mutants in which two to three contiguous, or near-contiguous, conserved residues were changed to alanine. This screen yielded three lethal mutants, IYY347–48+350AAA, LLF360+362–63AAA, and ITR378–380AAA, and six mutants with slow growth phenotypes, LIV339–341AAA, RF369–370AA, GIT390–392AAA, QII395–397AAA, YL399–400AA, and DER439+447+450AAA.

To refine the residues that are important for Tfb2 function, we performed a third screen in which we created single and double alanine mutants involving the residues found to be required for normal growth in the triple mutants. Four of the double alanine mutants, IY347-348AA, YY348+350AA, IT378-379AA, and II396-397AA, yielded growth phenotypes. Interestingly, amongst the 53 single alanine substitution mutants, only F370A yielded a slow growth phenotype and it was mild. This result indicates that HubA function is largely unaffected by the loss of single amino acid side chains from conserved residues.

In addition to the alanine substitution mutants, we created the following mutations in Tfb2: E342R, F, and Q, E342R+K346E, D439K, E447K, and R450E. The E342R and E342R+K346E mutants were created based on the finding that human p52 variants, containing radical mutations at the equivalent positions, are defective for interaction with XPB, and flies containing the homozygous E310 (equivalent to E342 in yeast) to K mutation are inviable (24). As expected, both mutants, as well as the E342F mutant, were also lethal in yeast. The only other mutant amongst this group that yielded a growth phenotype was E447K.

In total, we created 90 mutant *TFB2* alleles, amongst which six were lethal, and 12 displayed slow growth phenotypes in single colony growth assays (Table 1). Among the conditional mutants, the strains expressing the RF369–70AA, YY348+350AA, IT378–79AA, and YL399–400AA alleles displayed the most severe growth phenotypes at 37 °C.

**Table 1:** Summary of phenotypes for the Tfb2 HubA mutants identified in this study

Mutants whose growth phenotypes are suppressed by TFB6 are highlighted in yellow. Mutants whose growth phenotypes are not suppressed by TFB6 are highlighted in orange.

**Figure 2:**
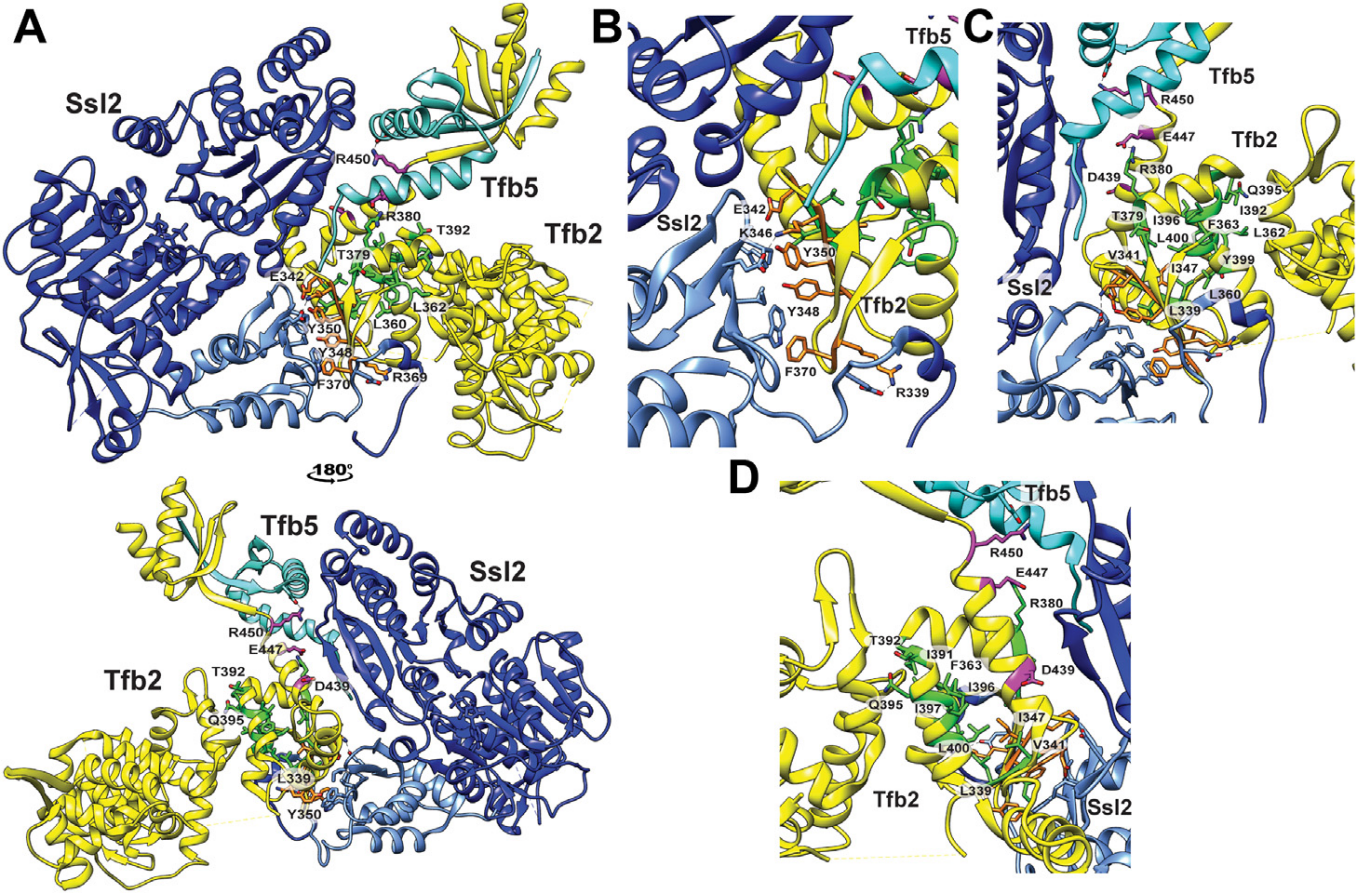
**Classification of Tfb2 HubA mutants.***A*, Tfb2 HubA mutations mapped onto Tfb2 in the cryo-EM model of contracted TFIIH in the PIC (Protein Data Bank ID: 7O4K). Mutation of Tfb2 residues corresponding to group 1, 2, and 3 mutants are colored *orange*, *green*, and *magenta*, respectively. *B*, close-up view of group 1 mutants (*orange*). *C* and *D*, close-up view of group 2 (*green*) and 3 mutants (*magenta*). Only Ssl2 (*blue* and *light blue* for the Clutch domain), Tfb2 (*yellow*), and Tfb5 (*cyan*) are shown for clarity. PIC, preinitiation complex.

Mapping the mutations onto the recently published cryo-EM structures of TFIIH revealed that they clustered into three regions (Fig. 2, *A–D*). Group 1 mutations map to the Ssl2 Clutch–Tfb2 HubA interaction interface. Mutation of these residues is predicted to disrupt key hydrophobic or electrostatic interactions with Ssl2 residues at the Ssl2 Clutch–HubA interaction interface. Group 2 residues are largely hydrophobic and primarily map to internal regions of HubA. Loss of these side chains likely effects the folding of the HubA domain. Group 3 mutations (E447K and DER439+447+450AAA) map to the C-terminal region of the HubA domain bordering on the dimerization domain. hE397/yE447 interacts with conserved hR348/yR380 in the second α helix of the HubA domain and hR400/yR450 interacts with conserved D13 in p8/Tfb5. Disruption of the hE397/yE447–hR348/yR380 interaction likely destabilizes the C-terminal region of p52/Tfb2, which in turn effects the ability of the p52/p8 dimer to interact with the second RecA domain of XPB. Disruption of the p52/Tfb2 hR400/yR450–p8/Tfb5 D13 interaction may also weaken the interaction between p52/p8 and the RecA domain of XPB by destabilizing the p52–p8 interaction.

### A genetic interaction between some *tfb2* HubA alleles and *TFB6*

To determine whether the Tfb2 HubA mutations affect cell growth in the presence of *TFB6*, we screened the set of mutants that displayed lethal or conditional growth phenotypes in a strain with a WT copy of *TFB6* (Table 1 and Fig. S3). Among the lethal Tfb2 HubA mutations tested, EK342+346RE, E342F, E342R, and IYY347–348+350AAA remained lethal in the *TFB6* background, whereas LLF360+362–363AAA, ITR378–380AAA displayed a mild slow growth phenotype at 37 °C. Among the mutants that displayed a slow growth phenotype in the absence of *TFB6*, the phenotypes of RF369–370AA, YY348+350AA, and DER439+447+450AAA were unchanged by the presence of *TFB6*, whereas strains carrying the LIV339–341AAA, IY347–348AA, IT378–379AA, YL399–400AA, GIT390–392AAA, QII395–397AAA, II396–397AA, F370A, and E447K alleles no longer displayed a slow growth phenotype in the presence of *TFB6*. Interestingly, the growth phenotypes of all group 2 HubA mutant alleles were relieved by *TFB6*. On the other hand, all the group 1 mutants, except for the weak F370A mutant, did not interact genetically with *TFB6*. The results show that group 2 mutants that map to internal regions of HubA require *TFB6* for normal growth, whereas most group 1 mutants that map to the Ssl2 Clutch–Tfb2 HubA interaction interface are independent of *tfb6*Δ. We also tested group 1 mutants, RF369–370AA and YY348+350AA, and group 2 mutants, IT378–379AA and YL399–400AA, which grew very slowly in the presence or the absence of *TFB6*, respectively, for UV sensitivity in the *TFB6+* background (Table 1). Group 1 mutants were very sensitive to UV, whereas group 2 mutants were not UV sensitive.

### Tfb2 HubA mutants are defective for interaction with Ssl2

**Figure 3:**
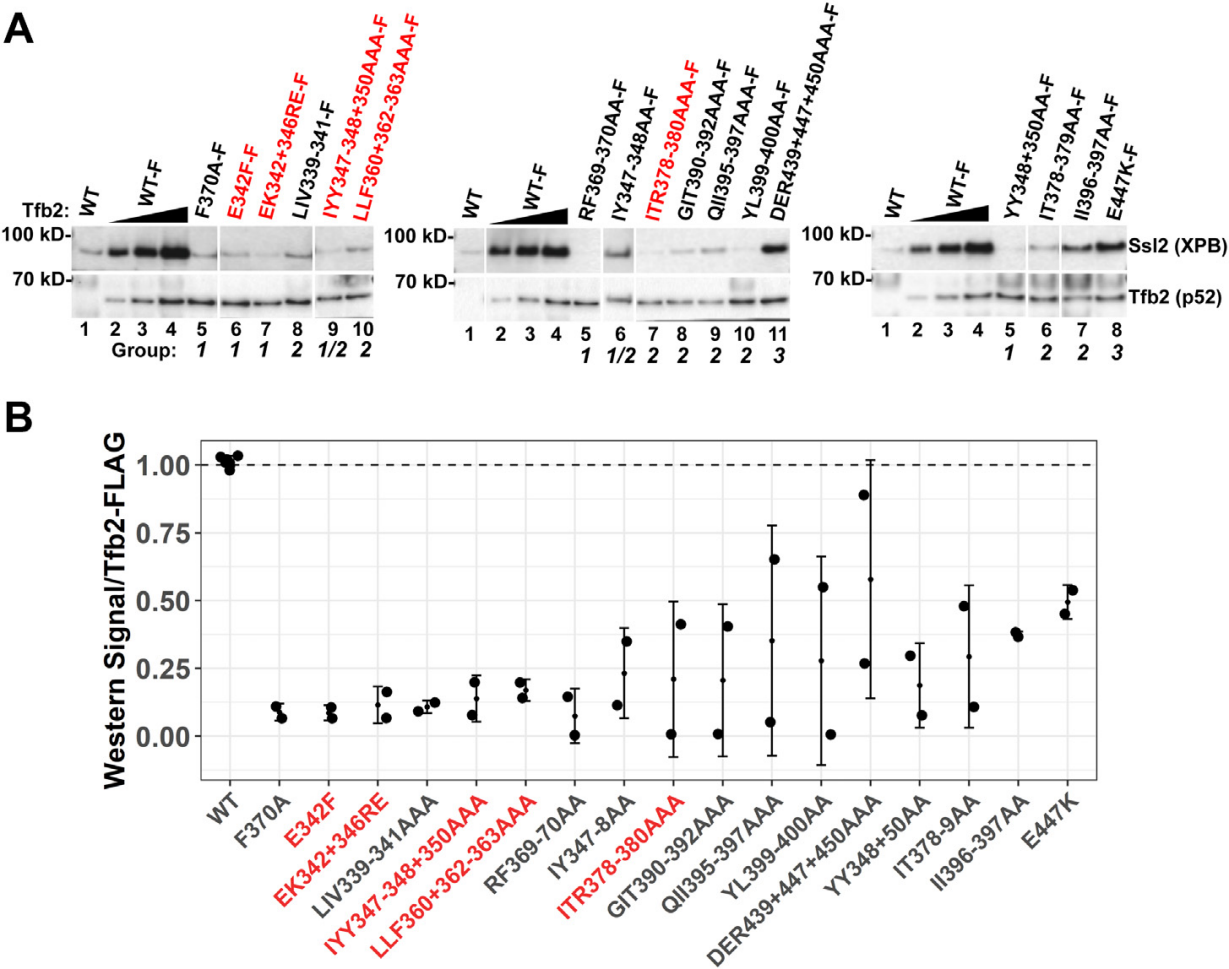
**Immunoprecipitation (IP) analysis of FLAG-Tfb2 HubA mutants.***A*, whole-cell extracts from strains expressing C-terminal 3× FLAG (F)-tagged WT Tfb2 or the indicated FLAG-tagged Tfb2 derivatives were subjected to IP and Western blot analysis using antisera against FLAG-Tfb2 and Ssl2. *Red font* indicates that cells expressing the mutant allele as the sole source of Tfb2 are inviable. The different groups of mutants are also indicated. For the mutants, the standard deviation based on two biological replicates is shown. *B*, quantitation of Ssl2 signal in WT and mutant Tfb2 IPs.

To examine the underlying biochemical defect of the Tfb2 HubA mutants, we performed IP analyses of both viable and inviable mutants. C-terminal FLAG-tagged Tfb2 variants were coexpressed with endogenous Tfb2. Whole-cell extracts (WCEs) from these strains were used to immunoprecipitate the mutant subunit *via* the FLAG tag, and association of the mutant subunit with Ssl2 was analyzed by Western blot (Figs. 3, *A* and *B* and S4). Consistent with predictions from the cryo-EM structure, all group 1 mutants failed to pull down Ssl2. Group 2 mutants displayed a range of interaction defects from mild to severe. Since the mutated residues do not make direct contacts with Ssl2 in the cryo-EM model, the defects may be a result of destabilized structure. Group 3 mutant E447K was mildly defective for Ssl2 interaction, perhaps a reflection of an altered ability of the Tfb2/Tfb5 dimer to interact with the second RecA domain of Ssl2.

### Human p52 HubA mutants are defective for interaction with XPB

**Figure 4:**
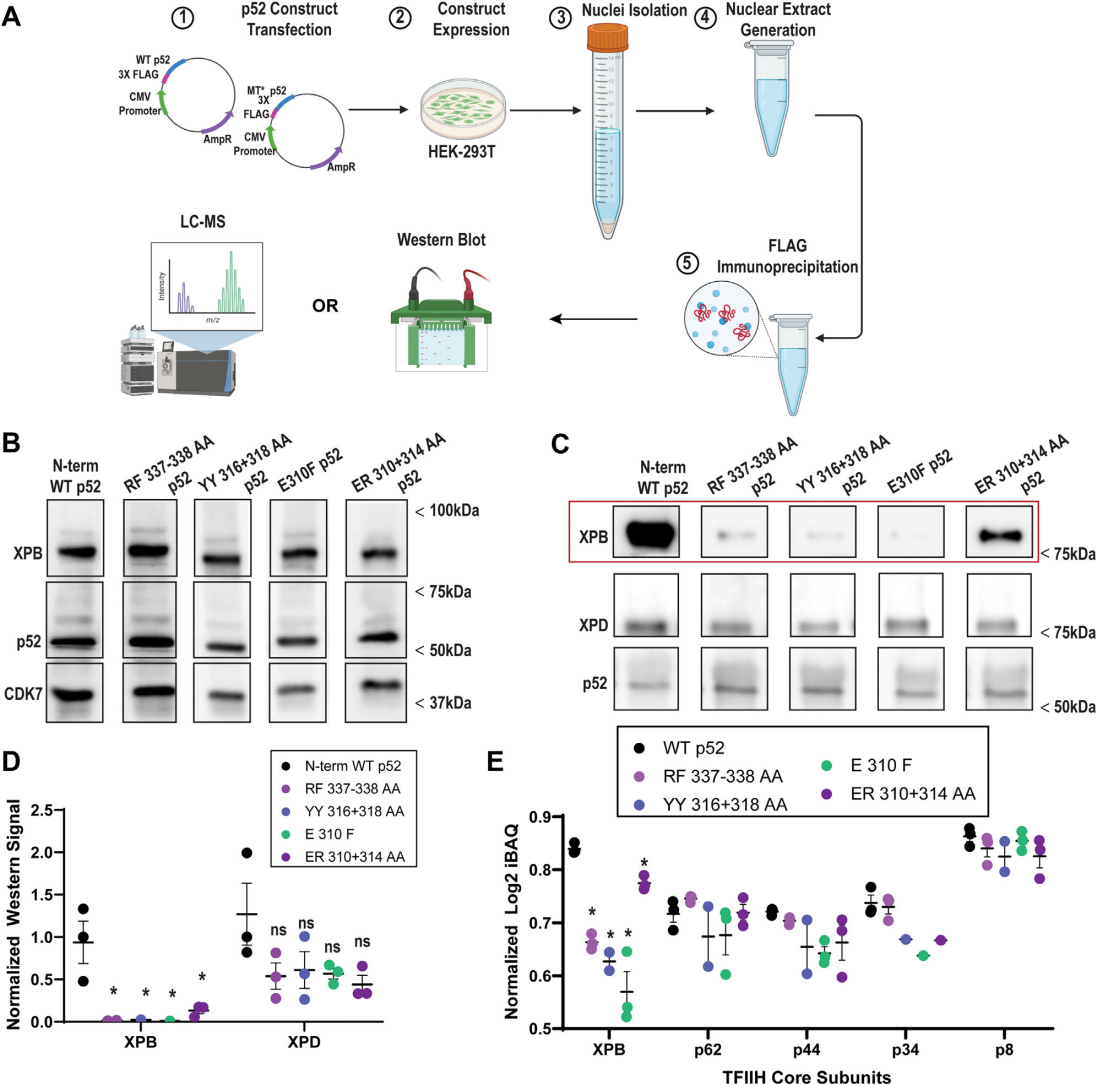
**Immunoprecipitation (IP) reveals that human XPB fails to associate with p52 HubA mutants.***A*, experimental overview; p52 expression constructs (WT or mutants RF337–338AA, YY316+318AA, ER310+314AA, E310F, each with N-terminal 3× FLAG) were transfected into HEK293T cells. Nuclear extracts were prepared and used for FLAG-IP–Western or LC–MS analysis. Schematic is made with BioRender. *B*, immunoblots of “input” nuclear extracts prepared from HEK293T cells. *C*, representative Western blots of anti-FLAG immunoprecipitated material from cells expressing the indicated FLAG-tagged p52 protein. Levels of XPB are decreased in p52 mutants (*red box*). *D*, quantitation of Western blot data from WT and mutant TFIIH complexes. For quantitation, band density was normalized to p52 WT signal. XPB abundance was significantly reduced in mutant complexes (padj = 0.006), but XPD abundance was not reduced with statistical confidence (ns; padj >0.05); n = 3, biological replicates. *E*, LC–MS results from anti-FLAG immunoprecipitated material; iBAQ (intensity-based absolute quantitation) results are shown comparing cells expressing WT p52 *versus* the indicated HubA mutants. XPB was significantly reduced in the HubA mutants (*p* < 0.05); specifically, RF337–338AA, padj = 0.0003; YY316+318AA, padj = 0.0002; ER310+314AA, padj <0.0001; and E310F, padj = 0.045. XPD and CAK subunits were not detected; n = 3, biological replicates. CAK, CDK-activating kinase; HEK293T, human embryonic kidney 293T cell line; padj, adjusted *p* value.

To further assess the impact of the Tfb2 mutations that resulted in severe Ssl2 association and growth defects, we introduced equivalent mutations into human p52. We transiently expressed N-terminal FLAG-tagged WT or mutant p52 in human embryonic kidney 293T (HEK293T) cells, followed by purification with an anti-FLAG affinity resin (Fig. 4*A*). The following mutant p52 constructs were tested: RF337–338AA, YY316+318AA, and E310F (equivalent to group 1 Tfb2 mutants RF369–370AA, YY348+350AA, and E342F, respectively). We also created an ER310+314AA p52 mutant construct, similar to group 1 Tfb2 mutant EK342+346RE. All constructs expressed well in HEK293T cells, with core and CAK subunits detected by Western blot (Fig. 4*B*). Immunoprecipitated FLAG-p52 TFIIH complexes were analyzed by Western blot (Figs. 4, *C*, *D*, S5, *A*, and *B*), which revealed a significant reduction of XPB in the mutant p52 complexes, consistent with results from yeast. While all tested mutants, RF337–338AA, YY316+318AA, E310F, and ER310+314AA, had significantly reduced XPB levels, XPD levels remained unchanged. IP–mass spectrometry (MS) analysis (Fig. 4*E*) showed a similar reduction in XPB association with the p52 mutants with nonsignificant changes in the other TFIIH core subunits detected (p62, p44, p34, and p8). XPD was not detected in these experiments. Together with the IP analyses in yeast (Fig. 3 and the following sections), these data indicate the importance of select p52 HubA residues for stable association of XPB/Ssl2 with p52/Tfb2 throughout evolution.

Interestingly, the CAK was not detected in the FLAG eluates from WT or mutant p52 IPs, by Western blot or IP–MS (Figs. 4*E* and S6*A*). Viewing the TFIIH structure, the p52 N terminus or the C terminus was not predicted to disrupt any protein–protein interface (Fig. S5, *C* and *D*). However, the N-terminal FLAG tag could potentially disrupt CAK-core TFIIH association, given that the CAK subunit CDK7 was identified in the flow-through fractions (Fig. S5*E*). Placement of the FLAG tag at the p52 C terminus yielded the same results (Fig. S5*E*); thus, the p52 N- and C-terminal FLAG tag appears to disrupt CAK module (containing CDK7) association with core TFIIH (containing p52) in human cells, in contrast with results from yeast.

### Integrity analysis of TFIIH

**Figure 5:**
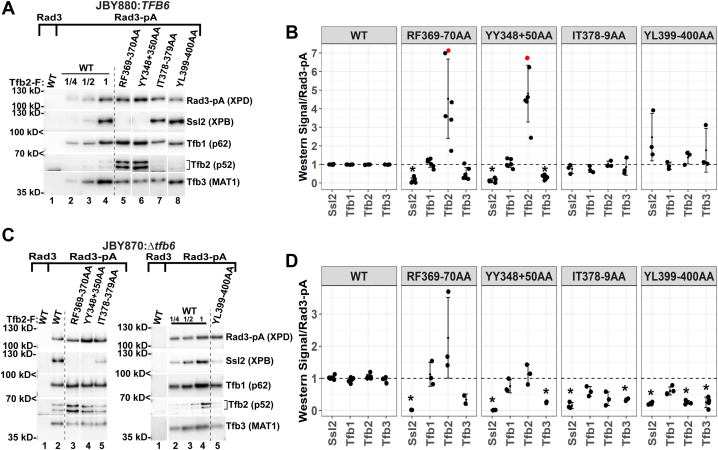
**TFIIH integrity analysis in strains expressing Tfb2 HubA mutants.***A*, whole-cell extracts from *Tfb6+* strains expressing the indicated FLAG-tagged Tfb2 derivatives and pA-tagged Rad3 were subjected to IP and Western analysis using antisera against the indicated TFIIH subunits. Extracts prepared from a strain expressing untagged Rad3 and FLAG-tagged WT Tfb2 were used as a control. *B*, quantitation of Western analysis. Significantly affected subunits are indicated by ∗ (padj <0.04). *Red dots* indicate data that correspond to the Western blots shown in *A*. *C*, whole-cell extracts from Δ*tfb6* strains expressing the indicated FLAG-tagged Tfb2 derivatives and pA-tagged Rad3 were subjected to IP and Western analysis using antisera against the indicated TFIIH subunits. Extracts prepared from a strain expressing untagged Rad3 and FLAG-tagged WT Tfb2 were used as a control. *D*, quantitation of Western analysis. Significantly affected subunits are indicated by ∗ (padj <0.04). IP, immunoprecipitation; padj, adjusted *p* value.

To evaluate the effect of the viable *tfb2* mutations on the integrity of TFIIH, we created strains expressing the mutant *tfb2* allele as the sole source of Tfb2 along with C-terminal protein A (pA)-tagged Rad3. We focused on group 1 and 2 Tfb2 mutations that had the strongest growth phenotypes in the presence (RF369–370AA and YY348+350AA) or the absence (RF369–370AA, YY348+350AA, IT378–379AA, and YL399–400AA) of *TFB6*. We created the strains in both *TFB6* and *tfb6*Δ backgrounds to gain insight into the possible biochemical basis underlying the genetic interaction between *TFB6* and group 2 HubA mutant alleles. Rad3-pA was immunoprecipitated with immunoglobulin G (IgG) beads from WCEs prepared from these strains, and the ability of TFIIH subunits to copurify with Rad3-pA was assessed by Western blot (Figs. 5 and S6). The effect of group 1 Tfb2 subunits, RF369–370AA, and YY348+350AA, on TFIIH integrity was similar in the presence (Fig. 5, *A* and *B*) and absence of Tfb6 (Fig. 5, *C* and *D*). Ssl2 failed to copurify with Rad3, CAK subunit Tfb3 levels were reduced in the YY348+350AA mutant, and Tfb1 was not significantly affected. Since the CAK is anchored to the core through interactions with both Rad3 and Ssl2 (17, 19), loss of Ssl2 in the YY348+350AA mutant IPs likely destabilizes the CAK. Tfb2 levels were elevated in some experiments, especially in the presence of Tfb6, but the result was not statistically significant because of the large variation in the estimated levels. It is possible that mutants RF369–370AA and YY348+350AA homodimerize in the absence of Ssl2 (20, 25). We note that the levels of all mutant Tfb2 subunits were elevated in WCEs derived from these strains (Fig. S7, *A* and *B*). In contrast, in IPs from cells expressing group 2 Tfb2 subunits, IT378–379AA and YL399–400AA, TFIIH integrity defects were only detected in the absence of Tfb6. Both Ssl2 and Tfb3 levels were reduced in IPs from both mutants, and Tfb2 levels were reduced in the YL399–400AA mutant. Interestingly, by quantitative MS analysis, we found that Tfb6 associates with TFIIH in IPs from the YL399-400AA mutant, whereas little to no Tfb6 was found in IPs from WT and the IT378–379AA mutant (Fig. S8). In addition, Tfb5 was not detected in the mutant IPs. Importantly, the ability of Ssl2 to associate with TFIIH correlates with the growth phenotypes of these mutants. Group 1 mutants grow slowly at elevated temperature in the presence and absence of Tfb6, and Ssl2 association with TFIIH is severely abrogated in both backgrounds. On the other hand, group 2 mutants only grow slowly in the absence of Tfb6, and Ssl2 association with TFIIH is compromised in this background.

As mentioned previously, Tfb2 protein levels are elevated in the mutant *tfb2* strains tested in this study, in both the presence and absence of Tfb6 (Fig. S7, *A* and *B*). It appears that yeast cells boost production of mutant Tfb2 subunits to try to compensate for defects in Tfb2 function. Furthermore, even in strains expressing WT Tfb2, loss of Tfb6 results in elevated Tfb2 levels (Fig. S9*A*), and ∼20 times more TFIIH is captured by Rad3-pA IP (Fig. S9, *B* and *C*). The elevated levels of TFIIH in the absence of Tfb6 may be due to both increased stability of holo-TFIIH (23) as well as increased production and/or stability of TFIIH subunits.

### Gene expression changes in *tfb2* HubA mutants

**Figure 6:**
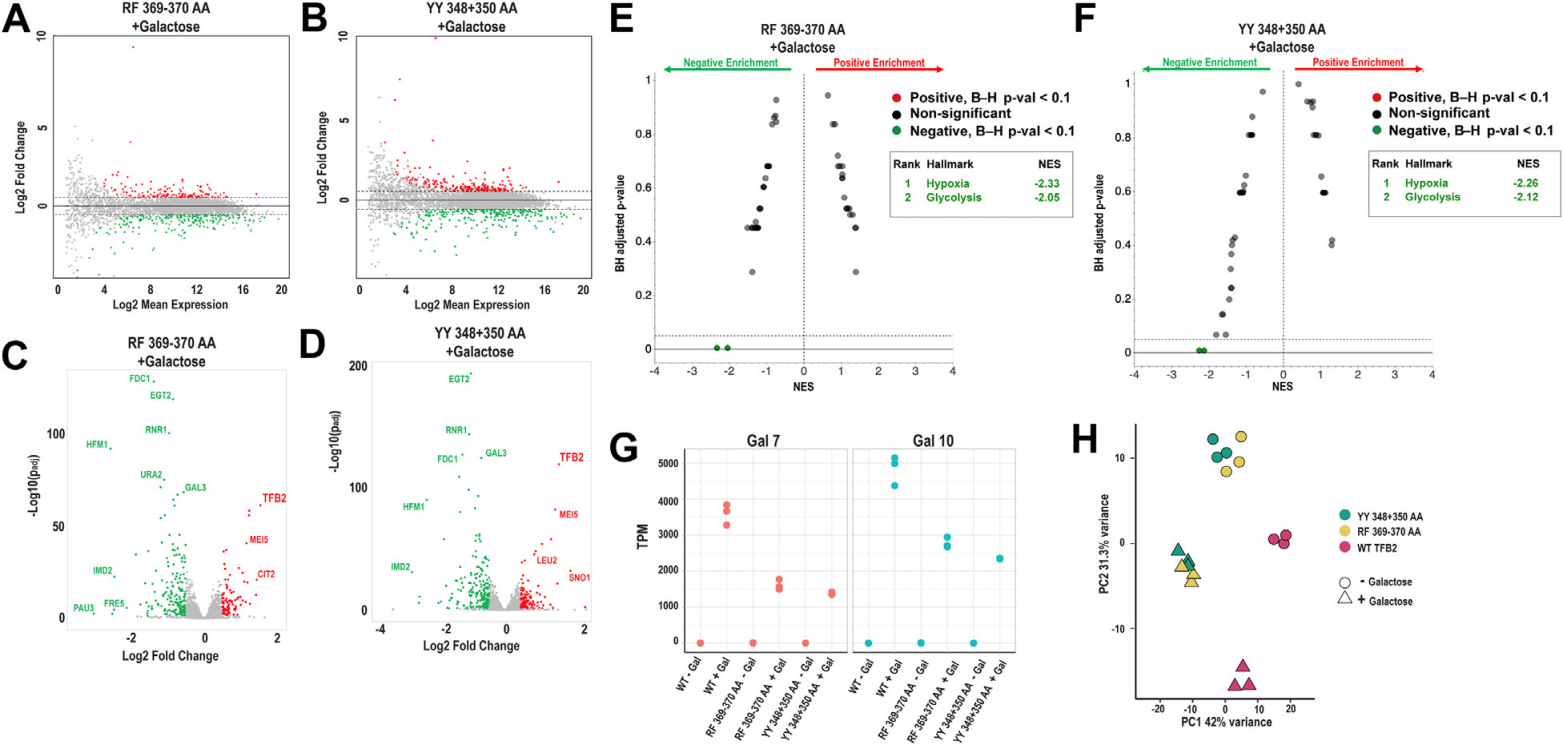
**RNA-Seq analysis of strains expressing Tfb2 HubA mutants following galactose induction.***A* and *B*, MA plots that show changes in gene expression in the yeast *tfb2* HubA mutants RF369–370AA (*A*) or YY348+350AA (*B*) compared with WT *TFB2* following 60 min galactose treatment. *Red dots* signify increased gene expression, and *green dots* represent decreased expression (padj <0.05 and absolute [log2 fold change] >0.5). *C* and *D*, volcano plots that show increased (*red dots*) and decreased (*green*) gene expression in *tfb2* HubA mutants after 60 min galactose treatment, compared with WT *TFB2* cells. *E* and *F*, gene set enrichment analysis (GSEA) comparing galactose-treated HubA mutants RF369–370AA (*E*) or YY348+350AA (*F*) *versus* WT *TFB2* cells. Pathways with Benjamini–Hochberg (BH) adjusted *p* < 0.1 are shown as *colored dots* and listed in the table (*inset*). *G*, the normalized gene expression (TPM; transcripts per kilobase million) for the *GAL7* and *GAL10* genes ± 60 min galactose induction, in WT *TFB2* cells and group 1 HubA mutants RF369–370AA and YY348+350AA. *H*, principal component analysis (PCA) indicates RNA-Seq data cluster predominantly by condition (±galactose) and *TFB2* genetic state (WT *versus* HubA mutants), with the mutants clustering together. NES, GSEA normalized enrichment score; padj, adjusted *p* value.

To gain additional insight into the functional impact of the two most severe conditional HubA alleles, we analyzed changes in the transcriptome of cells upon shifting the carbon source from raffinose to galactose, a perturbation known to induce expression of galactose-metabolizing genes as well as other genes (26). RNA was isolated from cells expressing WT Tfb2, the RF369–370AA mutant, or the YY348+350AA mutant, for RNA-Seq analysis. HubA mutants RF369–370AA and YY348+350AA both showed significant changes in gene expression (adjusted *p* value <0.05 and absolute [log2 fold change] >0.5) relative to WT Tfb2 in the presence or the absence of galactose with a preference for downregulation over upregulation (Figs. 6, *A*, *B*, S10, *A* and *B*). Mutant RF369–370AA had 288 genes significantly affected in the presence of galactose with 96 upregulated, including *TFB2*, and 192 downregulated, including *GAL3* (Fig. 6*C*). In comparison, mutant YY348+350AA had 333 genes significantly affected with 128 upregulated and 205 downregulated in the presence of galactose, with *TFB2* and *GAL3* similarly affected (Fig. 6*D*).

In the absence of galactose stimulation, both Tfb2 mutants showed fewer total affected genes compared with WT Tfb2, but with *TFB2* still upregulated; specifically, RF369–370AA had 59 upregulated and 181 downregulated genes (Fig. S10*C*) and YY348+350AA had 113 upregulated and 178 downregulated genes (Fig. S10*D*). Gene set enrichment analysis revealed both mutants, in the presence of galactose, exhibited a decrease in the metabolic hallmark pathways of glycolysis and hypoxia (Fig. 6, *E* and *F*). A similar trend was seen with these HubA mutants in the absence of galactose (Fig. S10, *E* and *F*); however, they also displayed an increase in oxidative phosphorylation genes. Comparisons of WT and mutant cells in their +Gal *versus* −Gal states revealed an overall induction of gene expression (Fig. S11, *A*, *D*, and *G*) with an emphasis on *GAL* genes (Fig. S11, *B*, *E*, and *H*) and the corresponding metabolic pathways (Fig. S11, *C*, *F*, and *I*). Of note, in the presence of galactose, *GAL7* and *GAL10* were so strongly differentially expressed in both mutants relative to WT that they were called as DESeq2 outliers because of a large Cook’s distance. However, comparison of the normalized gene expression (transcripts per kilobase million) values for both *GAL* genes following galactose induction in WT *versus* mutant cells revealed that both mutants failed to induce *GAL* genes to WT levels (Fig. 6*G*). Furthermore, principal component analysis demonstrated clustering of the data with 42% of the variance because of the genetic state of *TFB2* (WT *versus* HubA mutant) and 31.3% from the presence or the absence of galactose, validating data quality and reproducibility (Fig. 6*H*). Taken together, the RNA-Seq data show that cells expressing either HubA mutant are transcriptionally defective compared with WT cells, although each mutant upregulates expression of *TFB2*.

### Tfb2 HubA mutants are defective for PIC formation at *GAL* gene promoters

**Figure 7:**
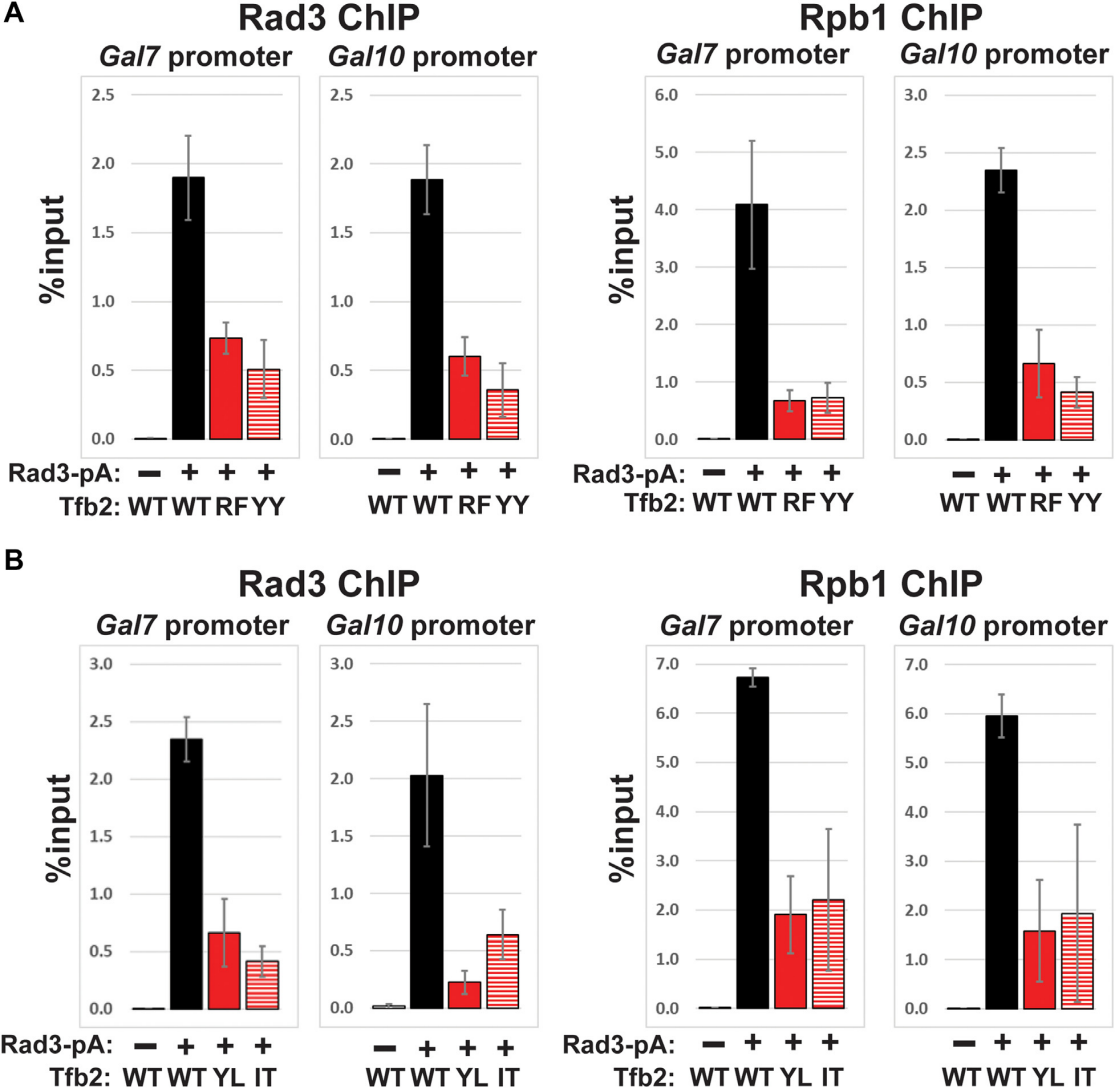
**ChIP analysis of Rad3 and Rpb1 at the *GAL7* and *GAL10* promoters after galactose induction.** In strains expressing group 1 (*A*) and group 2 (*B*) HubA mutants RF = RF369–370AA, YY = YY3+8+350AA, YL = YL399–400AA, and IT = IT378–379AA. ChIP, chromatin immunoprecipitation.

To gain insight into how group 1 HubA mutations RF369–370AA and YY348+350AA affect induction of *GAL* genes, we performed chromatin immunoprecipitation (ChIP) to monitor the ability of pol II and TFIIH to localize to *GAL* gene promoters after galactose induction. We also monitored the ability of pol II and TFIIH to localize to *GAL* gene promoters in strains expressing group 2 mutants IT378–379AA and YL399–400AA. The experiments were performed in the presence of *TFB6* for group 1 mutants and in the absence of *TFB6* for group 2 mutants. Pol II (Rpb1) and TFIIH (Rad3) localization to *GAL7* and *GAL10* promoters in both group 1 and 2 mutants was defective (Fig. 7, *A* and *B*). Combined with the results presented previously, these results show that mutations in the HubA domain disrupt TFIIH integrity and are defective for induction of *GAL* genes because of inefficient recruitment and/or stability of PIC components to *GAL* gene promoters.

### Tfb2 HubA mutants display altered transcription start site selection

**Figure 8:**
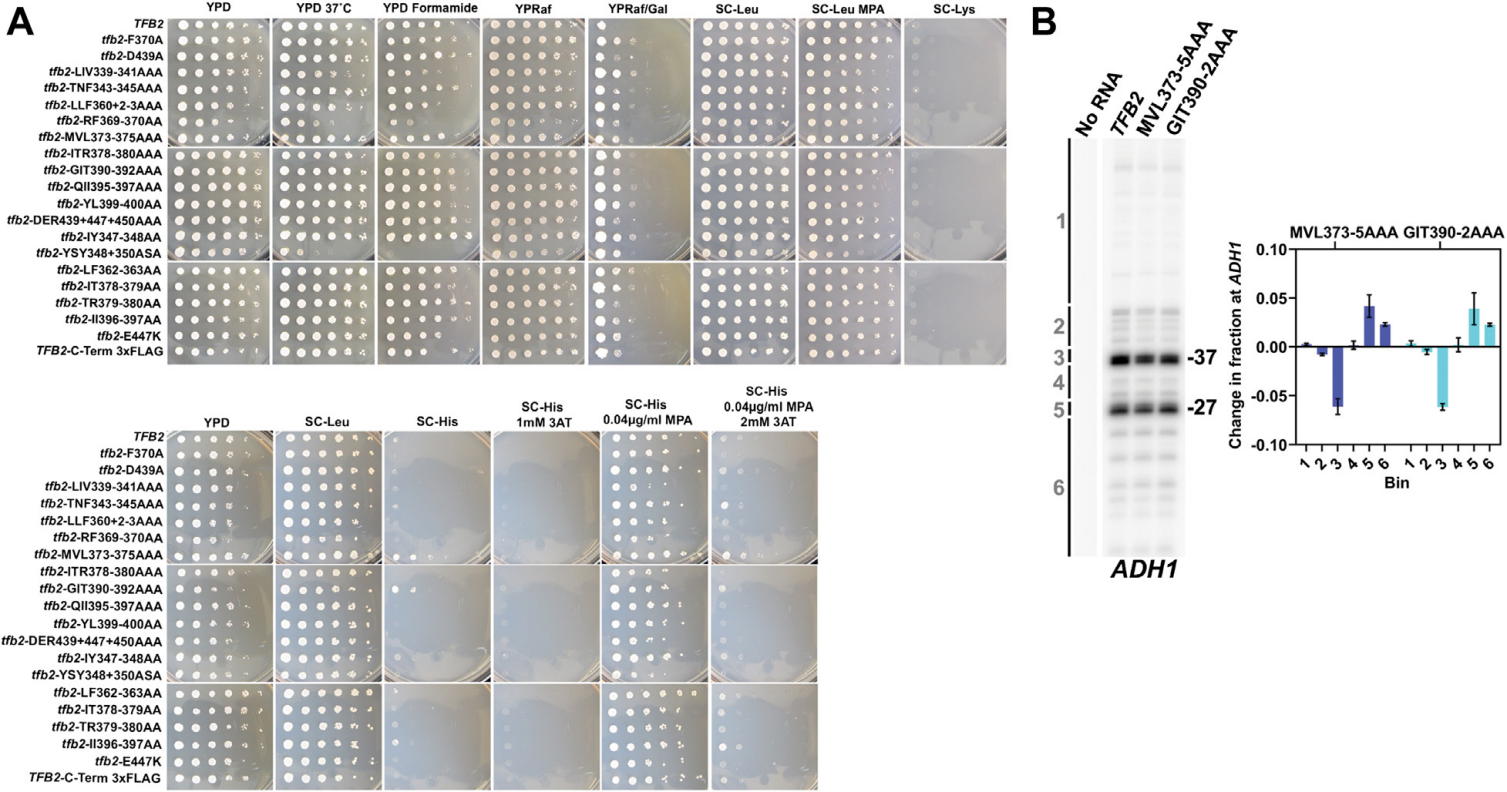
**Genetic and molecular defects of *tfb2* alleles in TSS usage.***A*, *tfb2* alleles were introduced into an *IMD2* background (*TFB6*) to examine general growth phenotypes or conditional phenotypes (*top panels*) or an *imd2Δ::HIS3* background (also *TFB6*) to detect defects in TSS shifts at the *IMD2* promoter (*bottom panels*). Phenotypes for the *IMD2* strain were growth on glucose (YPD), raffinose (YPRaf), or minimal medium (SC-Leu). Conditional phenotypes were increased temperature (YPD = 37 °C) or the presence of formamide (3%, YPD formamide). YPRaf/Gal detects transcription phenotypes at the *gal10Δ56* allele (see Zhao *et al.*). The presence of mycophenolic acid (20 μg/ml; SC-Leu MPA) can detect induction or TSS defects at *IMD2* (see main text). Phenotypes for the *imd2ΔHIS3* strain were controls (YPD or SC-Leu), SC-His to detect constitutive expression of *HIS3* under control of the *IMD2* promoter. SC-His with 1 mM 3-aminotriazole (3-AT) similarly detects constitutive expression of *IMD2* in the presence of a competitive inhibitor of His3 protein to assess if His+ phenotypes are strong (can grow in the presence of 3-AT) or weak (cannot). A small amount of MPA in the medium activates the *IMD2* promoter by inducing a TSS shift. Failure to do so would be consistent with defects in initiation that confer MPA sensitivity in an *IMD2+* background. *B*, primer extension analysis of *ADH1*. A primer downstream of *ADH1* was used for reverse transcription of *ADH1* mRNA for 5′ end analysis of *ADH1* transcripts. Products were separated by denaturing polyacrylamide gel electrophoresis (*left*). Products were quantified by binning (illustrated to *left* of gel) and normalization to total signal within each lane with WT bin signals subtracted from mutant to determine relative shifts. Primer extension reactions were from three biological replicates. mRNA purifications and error bars indicate standard deviation. MPA, mycophenolic acid; TSS, transcription start site; YPD, yeast extract–peptone–dextrose.

A subset of the *tfb2* HubA alleles were introduced into two strains allowing detection of transcription start site (TSS) shift phenotypes (Fig. 8*A*). One strain contains the *IMD2* promoter driving *HIS3* allowing phenotypes at the TSS-sensitive *IMD2* promoter to be detected (27, 28). Growth recapitulates defects of RF369–370AA and YY348+350AA as both are temperature sensitive here and exhibit sensitivity to formamide at 30 °C, consistent with a destabilization or protein folding defect (29). Of note, two *tfb2* alleles, MVL373–375AAA and GIT390–392AAA, both showed a weak His+ phenotype for the TSS-defect sensitive reporter. This phenotype relates to constitutive expression of *HIS3* driven by the *IMD2* promoter and is found in mutants that shift TSS usage to downstream positions (27, 28, 30). Zhao *et al.* previously identified Ssl2 substitutions at the Ssl2 Clutch–Tfb2 HubA interface conferring this phenotype that also showed downstream TSS shifts at *ADH1*, consistent with global shifts in TSS usage. To ask if *tfb2* His+ alleles showed similar TSS shifts at *ADH1*, we performed primer extension (Fig. 8*B*). As predicted, the weak *tfb2* His+ alleles showed downstream shifts in TSS usage at *ADH1* consistent with altered PIC activity and potential alteration to Ssl2 function. We also found that five *tfb2* HubA alleles (LIV339–341AAA, YY348+350AA, RF369–370AA, QII395–397AAA, and YL399–400A) were mildly sensitive to the drug mycophenolic acid (MPA) (Fig. 8*A*), a phenotype that is predictive of defects at the *IMD2* gene, especially upstream TSS shifts in certain pol II and GTF alleles (30, 31, 32, 33). As MPA sensitivity in *ssl2* mutants was predictive of upstream TSS shifts at *ADH1* and globally in yeast (27), we predict that *tfb2* mutants that are MPA sensitive confer defective TSS scanning.

## Discussion

Previously, based upon conserved protein–protein crosslinks and structural, biochemical, and genetic data, we proposed a model for the architecture of TFIIH (19). One prediction from this model was an interaction network involving the HubA domain of p52/Tfb2, the N- and C-lock domains of XPB/Ssl2, and p8/Tfb5. Here, we systematically mutagenized the HubA domain of Tfb2 and identified conserved residues that are required for normal growth of yeast cells in the absence of *TFB6*. Mapping these mutations onto the recent cryo-EM structures of TFIIH revealed that group 1 HubA mutations map to the predicted interaction interface between HubA and the XPB/Ssl2 Clutch; group 2 mutations are located within the HubA domain and likely affect the folding of p52/Tfb2; group 3 mutations are located at the C-terminal end of HubA and likely affect the ability of p52/Tfb2 and p8/Tfb5 to interact with the second RecA domain of XPB/Ssl2 (Fig. 2).

Next, we assayed the growth phenotypes of the *tfb2* mutants in a *TFB6* strain background. The slow growth/lethality of all group 2 mutants was relieved by *TFB6* (Fig. S3). Conversely, *TFB6* did not relieve the slow growth/lethality of most group 1 mutants nor the group 3 DER439+447+450AAA mutant. TFIIH integrity analysis revealed that while Ssl2 failed to stably interact with TFIIH in strains expressing group 1 RF369–370AA and YY348+350AA mutants in the presence and absence of *TFB6*, the ability of Ssl2 to associate with TFIIH was only affected in the absence of *TFB6* in strains expressing group 2 mutants YL399–400AA and IT378–379AA (Fig. 5). Biochemical analysis of human p52 with equivalent group 1 mutations also revealed an XPB interaction defect (Fig. 4), confirming the importance of these residues for the XPB–p52 interaction through evolution. Further characterization of group 1 and 2 mutants revealed that they were defective for recruitment of TFIIH and pol II to *GAL* gene promoters (Fig. 7), and we also showed that group 1 mutants were defective for induction of *GAL* genes (Fig. 6). Finally, we identified two *tfb2* HubA alleles that display altered TSS usage at the *ADH1* promoter (Fig. 8).

### Identification of functionally important HubA residues at the p52/Tfb2–XPB/Ssl2 interaction interfaces

Recent cryo-EM data reveal that p52/Tfb2 anchors XPB/Ssl2 to TFIIH *via* two main interfaces (5, 17). The p52/Tfb2 HubA domain contacts the XPB/Ssl2 Clutch and the p52/Tfb2–p8/Tfb5 dimer contacts the second RecA domain of XPB/Ssl2 (Fig. S12). The HubA domain and the XPB/Ssl2 Clutch assume homologous structures composed of four α-helices and five β-strands that interact by forming a pseudosymmetric dimer (17). The two domains interact through their β-sheets, *via* both hydrophobic and charged interactions. Our results establish the importance of specific, conserved hydrophobic, and charged residues within the p52/Tfb2 HubA domain for the interaction between p52/Tfb2 and XPB/Ssl2. The most severe XPB/Ssl2 interaction–defective mutations found in this study were E342R, E342F, EK342+346RE, RF369–370AA, and YY348+350AA. The Tfb2 E342 (E310 in human p52) mutants were lethal, and the importance of this residue for interaction with XPB/Ssl2 has been reported in humans, flies, and yeast (21, 24). E342 is part of a charge complementarity patch along with Tfb2 residue K346 (R314 in human p52) at the Tfb2–Ssl2 interface. Interestingly, substitution of alanine at either E342 or K346 is not detrimental for yeast growth, suggesting that the Tfb2–Ssl2 interaction can withstand loss of either side chain. Similarly, single alanine substitutions at Y348, Y350, R369, or F370 (Y316, Y318, R337, F338, respectively, in human p52) yielded no or mild (F370) growth phenotypes, but the combined YY348+350AA and RF369–370AA mutations yielded severe growth phenotypes in the absence of *TFB6*. R369 in *S. cerevisiae* Tfb2 interacts with a conserved aspartic acid in the NTD of Ssl2 and Y348, Y350, and F370 make conserved hydrophobic contacts at the HubA–Ssl2 Clutch interface. Group 3 DER439+447+450AAA (D389, E397, and R400 in human p52) mutation, located at the C-terminal end of HubA, likely affects the ability of Tfb2 to properly position the Tfb2–Tfb5 dimer for interaction with the RecA domain of Ssl2. This mutation may also destabilize the Tfb2–Tfb5 dimer, which in turn could weaken the interaction between the dimer and the second RecA domain of Ssl2.

While disease-associated mutations in TFIIH subunits XPB, XPD, and p8 have been known for some time (34), there are no reports of human diseases related to mutations in other TFIIH subunits. For p52, the only mutations described in multicellular organisms have been those isolated in flies and subsequently studied biochemically in a recombinant human system (24). These mutations include radical substitutions at E310 and R314, also studied in this report. Consistent with the results presented here, the E310K mutation affected the ability of p52 to interact with XPB. Interestingly, while the human p52 E310K/R314D mutant was defective for stable incorporation of XPB into TFIIH, the rest of TFIIH was unaffected. We observed that stable CAK association was often impacted when Ssl2 association with yeast TFIIH was destabilized (Fig. 5). Species-specific differences and/or the use of recombinant *versus* native expression could account for the apparent differences between the human and yeast data.

Recently, the Kisker group mutated a number of conserved residues in the HubA domain of *Candida thermophilum* p52 that are predicted to interact with the NTD of XPB and evaluated the ability of the mutant p52 subunits to interact with the NTD of XPB or to stimulate the ATPase activity of XPB (21). Mutation of residues equivalent to E342, E342/K346, Y348, and F370 and M373 in *S. cerevisiae* Tfb2 affected the ability of *C. thermophilum* p52 to interact with and to stimulate the ATPase activity of XPB. Our phenotypic and biochemical analyses of Tfb2 subunits containing mutations at these positions in *S. cerevisiae* also point to the importance of E342, Y348, and F370 for interaction with XPB/Ssl2 and TFIIH function, although for Y348, growth defects were only observed upon mutation of both Y348 and Y350. Interestingly, loss of conserved aromatic side chains appears to be most important for the p52/Tfb2–XPB/Ssl2 interaction.

### Impact of *tfb2* HubA mutations on PIC assembly and transcription

We observed that *tfb2* HubA mutations YY348+350AA and RF369–370AA that altered the ability of Ssl2 to stably associate with TFIIH led to defects in recruitment of pol II and TFIIH to *GAL* gene promoters after induction with galactose. As expected, these mutants were also defective for induction of *GAL* genes. The defects in PIC assembly and transcription because of loss of TFIIH function are consistent with results from studies that employed the anchor away technique (35) to deplete PIC factors from the nucleus (6, 36). Depletion of many PIC subunits, including core TFIIH subunits Ssl2, Rad3, and Ssl1, reduced the levels of other PIC factors and pol II at promoters and ORFs. Our results support and extend the results from these studies by showing that a normal Tfb2–Ssl2 interaction is required for proper PIC assembly and function *in vivo.* We note that these *in vivo* findings are unlike the situation *in vitro*, in which partial PICs containing pol II, but lacking TFIIH, can assemble (37, 38).

The Tfb2 mutants YY348+350AA and RF369–370AA each exhibit mild sensitivity to the drug MPA (Fig. 8*A*), predictive of TSS defects at the *IMD2* gene, whose promoter is exquisitely sensitive to TSS defects (39, 40). As MPA sensitivity in *ssl2* mutants is predictive of upstream TSS shifts at *ADH1* and globally in yeast (27), we predict that *tfb2* mutants that are MPA sensitive confer defective TSS scanning. These phenotypes also suggest that gene expression defects in the YY348+350AA and RF369–370AA mutants may arise from altered TSS usage post-PIC assembly and not only because of decreased levels of functional TFIIH in cells. Postpromoter melting phenotypes in promoter scanning, which requires Ssl2 translocation activity to reach downstream TSSs, suggest a network of interactions that regulate TFIIH activity in this process. Because p52/Tfb2 has been linked to regulation of XPB–Ssl2 ATPase activity, TSS defects may be due to TFIIH functional defects of the following types. First, alterations in Tfb2 stimulation of Ssl2 ATPase activity may cause changes in the rate of scanning. Second, alterations in Tfb2 stimulation of Ssl2 ATPase activity could affect Ssl2 translocation activity, which could affect Ssl2 processivity. Finally, changes in the Tfb2–Ssl2 interaction might affect Ssl2 processivity because of altered rates of conformational changes that could contribute to termination of Ssl2 translocation. Future studies will be needed to decipher the precise biochemical mechanisms.

### A genetic interaction between *TFB2* and *TFB6*

Tfb6 was discovered during purification of TFIIH from *S. cerevisiae* and was shown to be able to dissociate Ssl2 from TFIIH (23). In addition, the authors reported a 20-fold increase in the yield of holo-TFIIH in the absence of Tfb6. Deletion of *TFB6* had no discernable impact on cell growth in rich media and had no effects on transcription *in vitro* or gene induction *in vivo* (23). However, *TFB6* has both positive and negative synthetic interactions with a number of genes that encode subunits of TFIIH, including *SSL2*, *SSL1*, *TFB1*, and *TFB5* (23, 41, 42). In this study, we discovered a negative genetic interaction between group 2 *TFB2* HubA alleles and *TFB6*. Biochemically, Ssl2 association with TFIIH in cells expressing group 2 mutants IT378–379AA and YL399–400AA was defective only in the absence of Tfb6 (Fig. 5), and PIC formation was defective at *GAL* gene promoters (Fig. 7*B*). All group 2 mutations map to internal regions of HubA (Fig. 2), and likely alter the overall structure of Tfb2, affecting its ability to stably interact with Ssl2 in the absence of Tfb6. It is possible that defects in the Ssl2–Tfb2 interaction because of group 2 mutations are revealed in the absence of Tfb6 because Ssl2 is not subject to the dissociating activity of Tfb6 in this genetic background. These results raise the possibility that the destabilizing activity of Tfb6 plays a positive role in TFIIH function. Tfb6 might also suppress some group 2 mutants by more stably associating with TFIIH (Fig. S8).

We also found that loss of Tfb6 results in elevated levels of Tfb2 in cells expressing both WT and mutant Tfb2 subunits (Figs. S7 and S9*A*) as well as elevated levels of Rad3 and TFIIH, based on Western blot analysis of Ssl2, Tfb1, Tfb2, and Tfb3 in Rad3 IPs (Fig. S9, *B* and *C*). The elevated levels of TFIIH are consistent with observations by Murakami *et al.* (23). Since the protein abundances of Rad3 and Tfb2 are elevated in the absence of Tfb6, the increase in TFIIH levels may be due to both the loss of the Ssl2-dissociating activity of Tfb6 and an increase in the abundance of TFIIH subunits. Elevated TFIIH levels may be required for normal cell growth in the absence of Tfb6 because of loss of Ssl2 and/or Tfb6 activity in a post-transcriptional process. Ssl2 has been implicated in RNA export from the nucleus (43, 44). It is possible that Tfb6 facilitates Ssl2 and/or TFIIH function in post-transcriptional events, and, in the absence of Tfb6, elevated holo-TFIIH levels may counteract defects in these post-transcriptional processes to maintain appropriate levels of gene expression.

## Concluding remarks

In summary, we have identified evolutionarily conserved residues in the HubA domain of p52/Tfb2 required for the p52/Tfb2–XPB/Ssl2 interaction. The HubA mutations have the strongest effect on the p52/Tfb2–XPB/Ssl2 interaction map to the interaction interface with the NTD of XPB/Ssl2. Cells expressing these mutant Tfb2 subunits are defective for induction of *GAL* genes due, in part, to a defect in PIC formation at *GAL* gene promoters. Another group of HubA mutants that interact genetically with *TFB6* map to internal regions of the HubA domain. In *tfb6*Δ cells expressing this group of Tfb2 mutants, TFIIH integrity and PIC assembly at *GAL* promoters is defective, and the cells grow slowly compared with cells expressing WT Tfb2. Future studies will seek to understand the role of Tfb6 and its relationship to TFIIH.

## Experimental procedures

### Strains and genetic analysis

Yeast strains used in this study are listed in Table S2. Plasmid shuffle strains were transformed with plasmids containing *TFB2* point mutations and deletions, and transformants were selected on glucose complete synthetic medium minus leucine (CSM Leu^−^) at 30 °C. Three to nine independent transformants for each derivative were streaked onto CSM Leu− plates containing 5-fluoroorotic acid (5-FOA) at 30 °C to select for cells that had lost the WT *URA*^*+*^ plasmid and to test for viability of each construct in strains SHY906 and JBY772. Yeast strains that were 5-FOA resistant were tested for growth by spot assay on CSM Leu^−^ medium at 20, 30, and 37 °C. For analysis of *tfb2* mutants in CKY4136 and CKY4138, six independent transformants were patched to SC-Leu medium followed by replica plating and streaking on SC-Leu plus 5-FOA. Phenotyping and media for these strains followed conditions and media described by Zhao *et al.* (27).

### Plasmids

Deletion and point mutation constructs of *TFB2* derivatives are listed in Table S3. All Tfb2 constructs contain a tobacco etch virus (TEV) protease site followed by a 3× FLAG epitope at their C terminus.

### p52 FLAG IP and Western blot

HEK293T cells were grown to 70% confluency in 500 cm^2^ dishes, and Lipofectamine 2000 transfected with 148 μg DNA of N-terminal 3×-FLAG-tagged p52, C-terminal 3×-FLAG-tagged p52, and the following N-terminal 3×-FLAG-tagged p52 mutants: RF337–338AA, YY316+318AA, E310A+R314A, and E310F. Constructs were expressed for 48 h, cells harvested, and nuclei isolated to enrich for full 10-subunit TFIIH (45, 46). Nuclei were then resuspended in radioimmunoprecipitation assay containing protease inhibitors and nutated for 30 min 4 °C to lyse nuclear membrane. Benzonase was then added, and samples were nutated for 1 h at 4 °C to release any chromatin-bound complexes and samples were spun 13,000 rpm for 15 min at 4 °C to yield nuclear extracts. Nuclear extract protein concentrations were determined by Pierce Bicinchoninic Acid Assay and Western blot run with 10 μg total protein.

FLAG-M2 agarose beads (Sigma) were prepared by washing with 1 ml radioimmunoprecipitation assay buffer three times. Nuclear extracts were nutated with 100 μl of FLAG-M2 agarose beads for 3 h at 4 °C, washed four times with 0.5 M HEGN-containing protease inhibitors, washed twice with 0.15 M HEGN-containing protease inhibitors, and eluted with 1 mg/ml FLAG (Sigma) peptide in 0.15 M HEGN. Elutions were done as previously described for TFIIH purifications (45, 47).

Bead-bound and eluate material was then analyzed by Western blot. Western blot samples were run on a 4 to 20% gradient protein gel, transferred to a nitrocellulose membrane, blocked with 5% milk, cut, and incubated with the following primary antibodies XPB (Santa Cruz; catalog no.: sc-293), XPD (Abcam; catalog no.: ab54676), FLAG-p52 (Sigma; catalog no.: F3165), MAT1 (Abcam; catalog no.: ab169546), and CDK7 (Santa Cruz; catalog no.: sc-856). Quantitation and analysis were performed with ImageJ and GraphPad Prism 9 (GraphPad Software, Inc) with normalization to the FLAG-p52 signal. Statistical analysis of the Western data was performed with GraphPad Prism 9 multiple *t* tests Holm–Sidak method, α = 0.05 with *p*-adjusted values reported (47).

### LC–MS of p52 FLAG IPs

Bead-bound material was also evaluated by LC–MS. Immunoprecipitated proteins were denatured, reduced, and alkylated in 5% (w/v) SDS, 10 mM Tris(2-carboxyethylphosphine), 40 mM 2-chloroacetamide, 50 mM Tris–HCl, pH 8.5 boiling for 10 min. Proteins were then digested using the SP3 method (48). Carboxylate-functionalized speedbeads (Cytiva Life Sciences) were added, the addition of acetonitrile to 80% (v/v) caused the proteins to bind to the beads. The beads were washed twice with 80% (v/v) ethanol and twice with 100% acetonitrile. Proteins were digested in 50 mM Tris buffer, pH 8.5, with 0.5 μg Lys-C/Trypsin (Promega) incubated at 37 °C overnight rotating to mix. Tryptic peptides were desalted using the carboxylate-functionalized speedbeads with the addition of 95% (v/v) acetonitrile twice and then eluted with 3% (v/v) acetonitrile, 1% (v/v) trifluoroacetic acid, and dried in a speedvac. Tryptic peptide samples were suspended in 3% (v/v) acetonitrile, 0.1% (v/v) trifluoroacetic acid, and directly injected onto a reversed-phase C18 1.7 μm, 130 Å, 75 mm × 250 mm M-class column (Waters), using an Ultimate 3000 UPLC (Thermos Scientific). Peptides were eluted at 300 nl/min using a gradient from 2% to 20% acetonitrile over 40 min into a Q-Exactive HF-X mass spectrometer (Thermo Fisher Scientific). Precursor mass spectra (MS1) were acquired at a resolution of 120,000 from 380 to 1580 *m/z* with an automatic gain control (AGC) target of 3E6 and a maximum injection time of 45 ms. Dynamic exclusion was set for 25 s with a mass tolerance of ±10 ppm. Precursor peptide ion isolation width for MS2 fragment scans was 1.4 Da, and the top 12 most intense ions were sequenced. All MS2 sequencings were performed using higher energy collision dissociation at 27% normalized collision energy. An AGC target of 1E5 and 100 ms maximum injection time was used. Raw files were searched against the UniProt Human database (UP000005640) using Maxquant (version 1.6.14.0) with cysteine carbamidomethylation as a fixed modification. Methionine oxidation and protein N-terminal acetylation were searched as variable modifications. All peptides and proteins were thresholded at a 1% false discovery rate and intensity-based absolute quantitation values reported. For visualization, the log2 of the intensity-based absolute quantitation values was displayed after normalizing to p52 abundance to account for sample concentration variability. Statistical analysis of the IP–MS was performed with GraphPad Prism 9 multiple *t* tests Holm–Sidak method, α = 0.05 with *p*-adjusted values reported (47).

### Tfb2 FLAG IP and Western blot analysis

About 200 ml of each yeast strain expressing both a FLAG-tagged Tfb2 derivative as well as WT Tfb2 was grown to an absorbance of 0.8 to 1 at 600 nm in CSM Leu−. Next, cells were harvested by centrifugation and washed with 1 ml of cold extraction buffer (EB; 50 mM Hepes [pH 7.6 at 4 °C], 250 mM potassium acetate [KOAc], 1 mM magnesium acetate [MgOAc], 1 mM EDTA, 20% glycerol, and 1 mM DTT with protease inhibitors) before being frozen in a dry ice bath and stored at −80 °C. Cells were resuspended in ∼1.5 ml cold EB and beaten with ∼0.7 ml glass beads in a Fisher Vortex Genie2. Cell debris was removed by centrifugation at 14,000*g* at 4 °C for 20 min. Protein concentrations were determined using the Pierce Bicinchoninic Acid Assay.

FLAG-M2 agarose beads (Sigma) were prepared by washing with EB, followed by incubating twice in 1 ml of 0.1 M glycine (pH 3.5) for a total of less than 5 min and washed three times in 1 ml of EB. Extracts were incubated with 15 μl of FLAG-M2 agarose beads for 2 h at 4 °C and then washed beads twice with 1 ml of EB and then with 1 ml of EB-N1 (EB containing 0.0025% Nonidet P-40, 150 mM KCl, and no protease inhibitors). Proteins were eluted by incubating beads twice in 20 μl of EB-N containing 0.15 mg ml^−1^ triple FLAG-epitope peptide (Genscript) for 30 min each at 4 °C.

Eluates were analyzed by Western blotting using polyclonal rabbit antibodies against Ssl2 (Hahn laboratory) and an anti-FLAG antibody (Sigma). Protein signals were visualized with a BioRad ChemiDoc XRS and quantified with Image Lab 5.2.1 software (Bio-Rad Laboratories). The abundance of each subunit was calculated based on a linear regression model of the signal from the WT IP (three or more data points) when the intensity of the subunit fell within the range of the linear model and the *r*-squared value was ≥0.85. Otherwise, the abundance of the subunit was determined based on its signal intensity relative to that of the subunit in the WT IP. The abundance of Ssl2 was normalized by the amount of Tfb2 in each eluate.

### Rad3–pA IP and Western analysis

Extracts and affinity beads were prepared as described for the Tfb2 FLAG IPs. About 0.5 mg IgG-conjugated Tosyl-Dynabeads (Thermo Fisher Scientific) were incubated with the 4 mg WCE prepared from strains expressing pA-tagged Rad3 and FLAG-tagged Tfb2 derivatives at 4 °C overnight. Beads were washed twice with 1 ml of EB, followed by three washes with 1 ml of EB-N2 (EB containing 0.05% Nonidet P-40, 150 mM KCl, and no protease inhibitors). Proteins were eluted proteins by incubating beads in 20 μl of EB-N2 containing 15 ng/μl TEV (UBPBio) for 2.5 h with shaking at 23 °C, followed by a second incubation in 20 μl of EB-N2 containing 15 ng/μl TEV for 2 h. Eluates were analyzed by Western blotting using polyclonal rabbit antibodies against Rad3 (Prakash laboratory), Ssl2, Tfb1, and Tfb3 (Hahn laboratory), Kin28 (PRB-260C; Covance), and Tfb2 (Ranish laboratory). Eluted proteins were quantified by Western blot and analyzed as described previously.

### LC–MS analysis of Rad3–pA IPs

Rad3–pA IPs were performed as detailed previously with the following changes. About 25 mg WCE and 2.5 mg IgG-conjugated Tosyl-Dynabeads were used per IP. Bead-bound proteins were eluted by incubating the beads in 33 μl of EB-N2 containing 45 ng/μl TEV for 2.5 h with shaking at 23 °C, followed by a second elution for 2 h, and a final wash. By volume, 75% of the eluates were prepared for MS analysis using S-Traps (ProtiFi; micro column) following the manufacturer's protocol. Samples were digested with 1 μg trypsin (Pierce) for 1 h at 47 °C. After drying the samples in a speedvac, they were resuspended in 3% acetonitrile, 0.1% formic acid, and injected onto a reversed-phase C18 3 μm, 100 Å, 75 μm × 200 mm trap column and 3 μm, 100 Å, 75 μm × 15 cm analytical column in series using an Easy nLC1000 (Thermo Fisher Scientific). Peptides were eluted at 300 nl/min using a gradient from 3 to 30% acetonitrile over 45 min and 30 to 45% acetonitrile over 15 min into an Orbitrap Eclipse mass spectrometer (Thermo Fisher Scientific). MS1 was acquired at a resolution of 120,000 from 375 to 1500 *m/z* with an AGC target of 1E6 and a maximum injection time of 100 ms. Duty cycle was set to 3 s with the most intense precursor ions selected for MS2 analysis in the ion trap with an AGC target of 1E4, maximum injection time of 50 ms, 30% normalized collision energy, and scan rate set to rapid. Precursor peptide ion isolation width for MS2 fragment scans was 1.6 Da, with dynamic exclusion enabled after two MS2 scans for 90 s with a mass tolerance of ±10 ppm. The identification, quantification, and statistical analysis were performed as detailed for the p52-IPs with the following exceptions. Raw files were searched against a custom UniProt database (2022) containing the *S. cerevisiae* proteome, TEV, rabbit-IgG, and trypsin using Maxquant (version 2.0.3.0). Statistical analysis was performed with R software.

### RNA-Seq analysis

RNA was prepared from cells expressing WT Tfb2, RF369–370AA, and YY348+350AA mutants after growth in CSM raffinose or after shifting the carbon source to 2% galactose for 60 min using a hot acidic phenol method (49). Samples were treated with DNAse I (Thermo Fisher Scientific) as recommended. Samples quality was assessed on the BioAnalyzer, and libraries were prepared from 1 μg RNA using the KAPA mRNA HyperPrep Kit. Equal amounts of the samples were pooled and sequenced on the NextSeq using the single-end 75 bp cycle (1 × 75).

Raw fastqs were evaluated for quality, adapters trimmed with cutadapt, version 1.16, and gene expression quantified using the pseudo-aligner salmon, version 0.10.2 with SacCer3 ensembl annotations. Principal component analysis was performed using the basic R stats package. MA plots were generated using the helper function of DESeq2 and depict a scatter plot of log2 fold changes (on the *y*-axis) *versus* the mean of normalized counts (on the *x*-axis). Differential gene expression analysis was performed using DESeq2, and differential genes called as adjusted p value <0.05 and absolute (log2 fold change) >0.5. Gene set enrichment analysis was done using the fgsea R package to utilize the log2 fold change as a rank metric for all genes relative to the Hallmark gene sets database for enrichment to determine which pathways were significantly affected, Benjamini–Hochberg (BH)–adjusted *p* <0.1.

Normalized bigwigs were generated by first aligning the trimmed fastqs to the SacCer3 reference genome using STAR to obtain .bams. The .bams were indexed using samtools, total read depth normalized, bed graphs were then generated with bedtools using these. bams, and the bed graphs were converted to bigwigs using University of California Santa Cruz’s bedGraphtoBigWig function.

### Primer extension analysis

Primer extensions were performed exactly as those described (27) adapting a protocol (50).

### ChIP

ChIP was performed essentially as described (51) with the following modifications. About 100 ml of yeast strains expressing WT Tfb2 or Tfb2 mutants were grown to an absorbance of 0.8 at 600 nm in CSM Leu− and 2% raffinose at 30 °C. For *GAL* gene induction, galactose was added to 2% followed by a 60 min incubation at 30°. Cells were crosslinked by addition of formaldehyde to 2%. Chromatin was sheared by sonicating with a Covaris S220 to yield an average DNA fragment size between 200 and 1000 bp. To measure Rad3–pA localization, sheared chromatin was incubated with IgG-conjugated Tosyl-Dynabeads for 3 h at 4 °C. To measure Rpb1 localization, sheared chromatin was incubated with anti-Rpb1 antibody 8WG16 (BioLegend) for 2 h at 4 °C followed by incubation with Protein G Dynabeads (Thermo Fisher Scientific) for 20 min at 23 °C. To quantify ChIP signals, quantitative PCRs (10 μl) were carried out with the appropriate primers (0.2 μM) and 1 U Taq. About 6.6% of the immunoprecipitated DNA and 0.06% of the input DNA were used as templates for the quantitative PCR. After 20 s at 95 °C, reactions were cycled 40 times at 95 °C for 1 s, 60 °C for 20 s, followed by a melt curve from 95 °C for 15 s to 60 °C for 60 s and 95 °C for 15 s. Relative ChIP signals were calculated by the percent input method. The location of the PCR products with respect to the translation start sites were *GAL10* promoter −204 to −37 bp, *GAL7* promoter −153 to +12 bp.

## Data availability

RNA-Seq data are deposited at Gene Expression Omnibus, GSE196069. The MS proteomics data for the Rad3 and p52 IPs have been deposited to the ProteomeXchange Consortium via the PRIDE partner repository with the dataset identifiers PXD035576 and PXD036107, respectively.

## Supporting information

This article contains supporting information (17).

## Conflict of interest

D. J. T. is a member of the SAB at Dewpoint Therapeutics. All the other authors declare that they have no conflicts of interest with the contents of this article.
