# Supplementary material for: Systematic mutagenesis of TFIIH subunit p52/Tfb2 identifies residues required for XPB/Ssl2 subunit function and genetic interactions with TFB6
Source: J Biol Chem. 2022 Aug 28;298(10):102433. doi: 10.1016/j.jbc.2022.102433 (PMC9557730; doi:10.1016/j.jbc.2022.102433)
Supplement: Supporting information [file mmc1.docx]

**Supporting information: 12 Supporting Figures with legends**

**Systematic mutagenesis of TFIIH subunit p52/Tfb2 identifies residues required for XPB/Ssl2 subunit function and genetic interactions with *TFB6***

Jacob Bassett^1^, Jenna K. Rimel^2^, Shrabani Basu^3^, Pratik Basnet^4^, Jie Luo^1^, Krysta Engel^5^, Michael Nagel^2^, Alexander Woyciehowsky^2^, Christopher C. Ebmeier^2^, Craig D. Kaplan^4^, Dylan J. Taatjes^2^, Jeffrey A. Ranish^1*^

^1^Institute for Systems Biology, Seattle, WA, USA

^2^Department of Biochemistry, University of Colorado, Boulder, CO, USA

^3^Department of Cell Biology, University of Pittsburgh, Pennsylvania, USA

^4^Department of Biological Sciences, University of Pittsburgh, Pittsburgh, PA, USA

^5^Boulder BioConsulting, Inc. Boulder, CO, USA

*corresponding author. [jeff.ranish@isbscience.org](mailto:jeff.ranish@isbscience.org)

Running title: Systematic mutagenesis of the HubA domain of p52/Tfb2

Keywords: general transcription factor TFIIH, p52/Tfb2, XPB/Ssl2, GTF2H4, Tfb6, RNA polymerase II, structure, function, mutagenesis, integrity, transcription start site

**
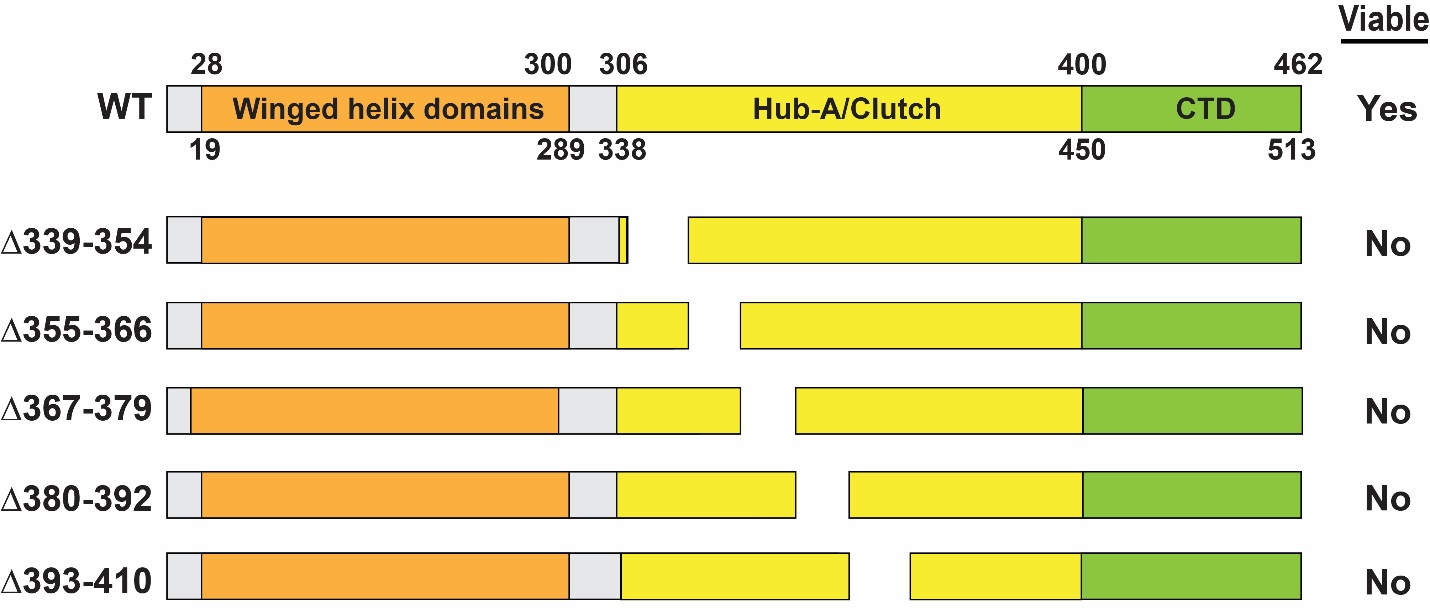
**

**Figure S1. Tfb2 HubA deletions.**

Schematic of Tfb2 domains and deletion derivatives. All deletion mutants were inviable.

**
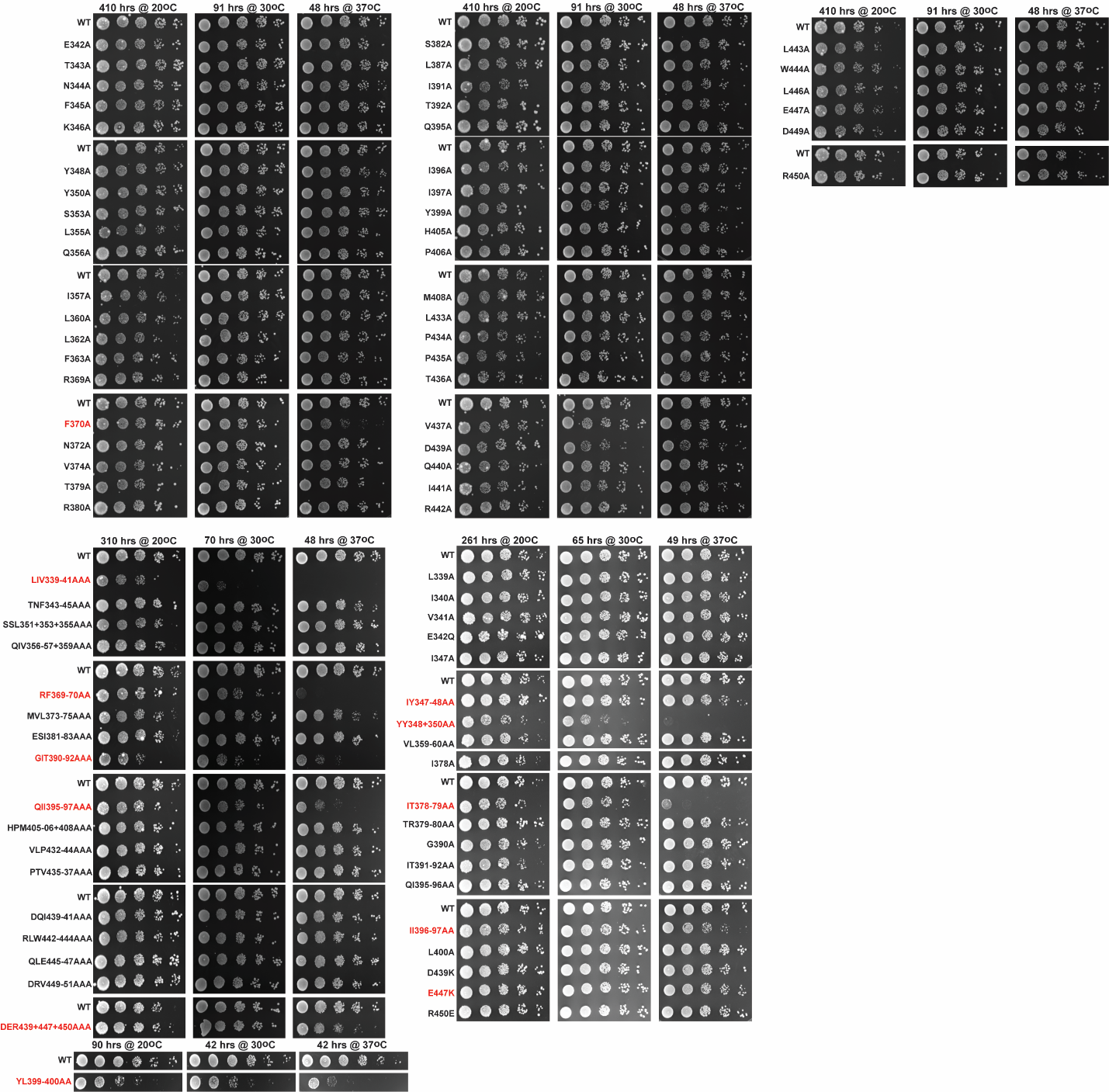
**

**Figure S2. Growth phenotypes of *tfb2* HubA mutants.**

Ten-fold dilutions of logarithmically growing yeast cells expressing the indicated *TFB2* alleles were spotted onto the CSM –Leucine medium and incubated at 20, 30, or 37ºC for the indicated times. Mutants that displayed a consistent slow growth phenotype are indicated by red font.

**
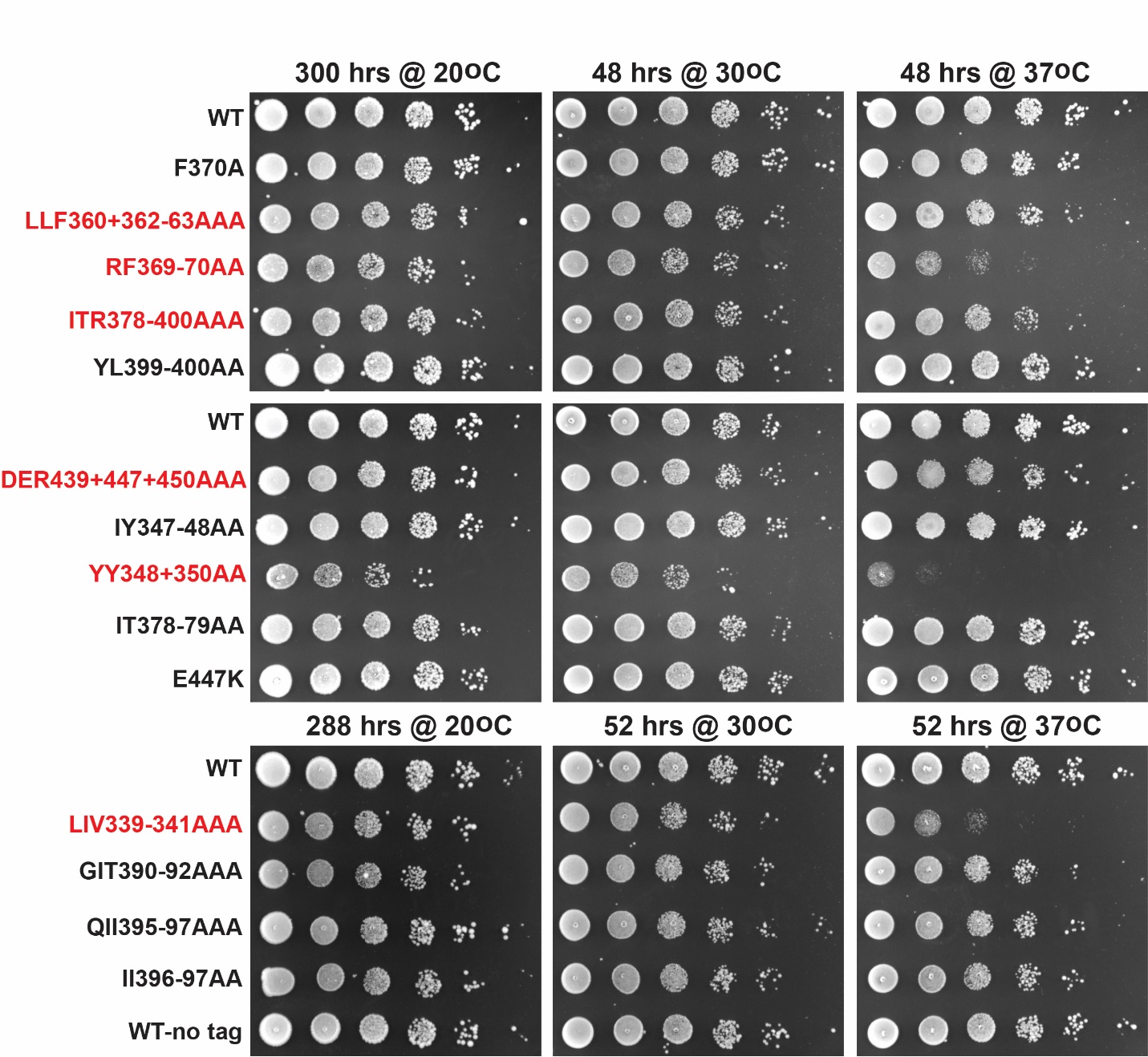
**

**Figure S3. Growth phenotypes of *tfb2* HubA mutants in a *TFB6* background.**

Ten-fold dilutions of saturated cultures expressing the indicated *TFB2* alleles were spotted onto the CSM –Leucine medium and incubated at 20, 30, or 37ºC for the indicated times. Mutants that displayed a consistent slow growth phenotype are indicated by red font.

**
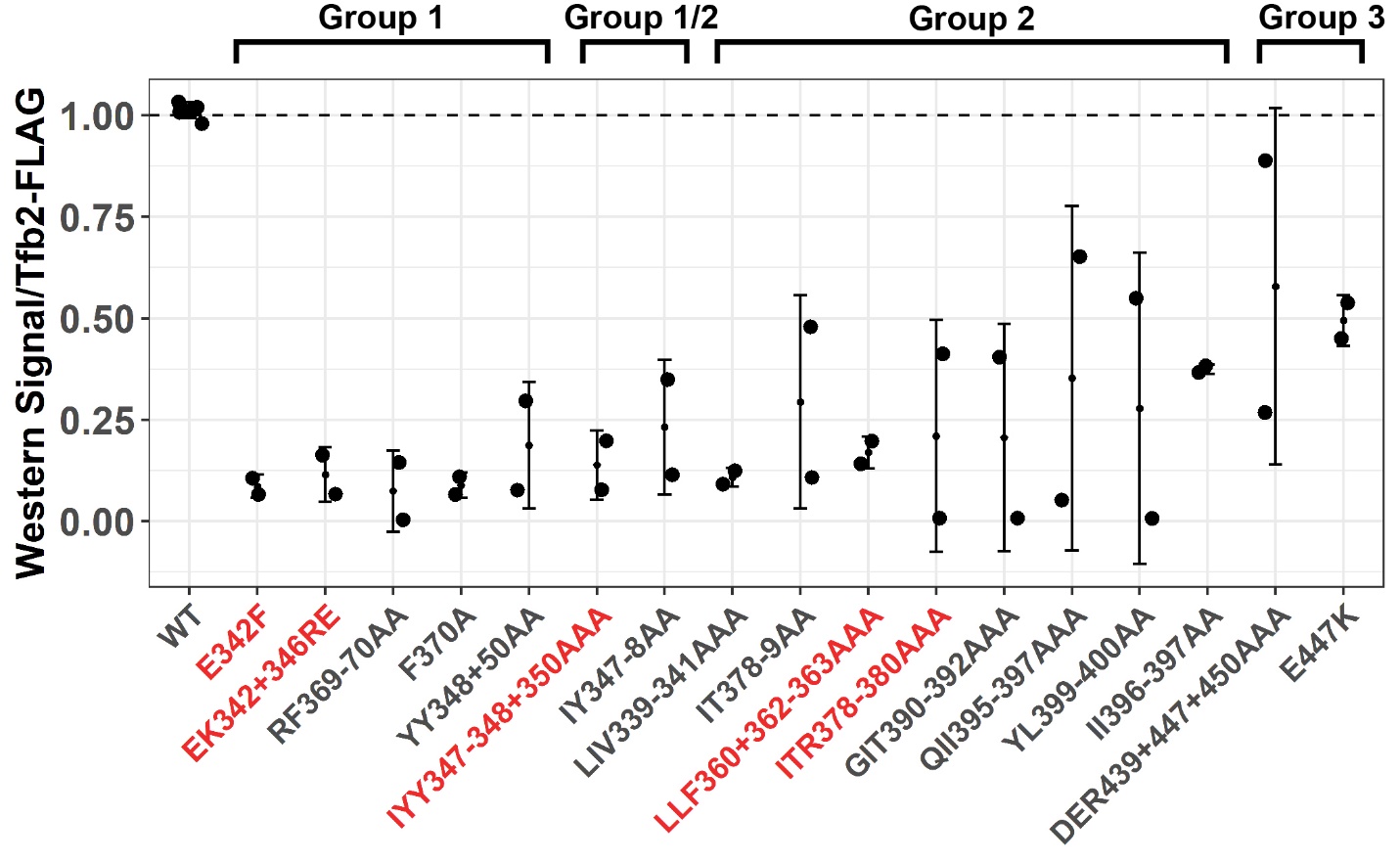
**

**Figure S4. Results of FLAG-Tfb2 immunoprecipitation analysis presented in Figure 3 arranged by mutant groups.**

Red font indicates that cells expressing the mutant allele as the sole source of Tfb2 are inviable. For the mutants, the standard deviation based on two biological replicates is shown.

**
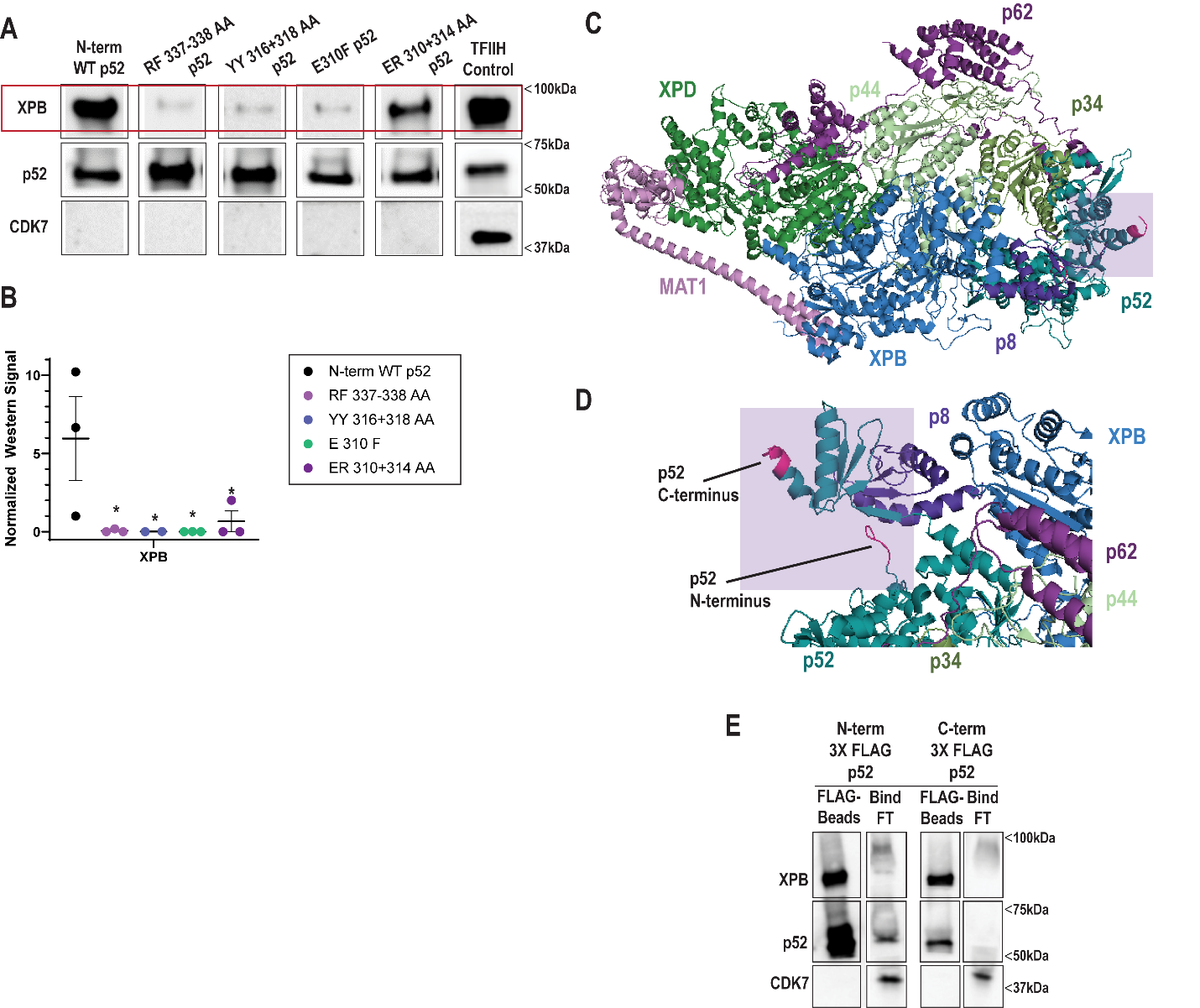
**

**Figure S5. Additional FLAG-p52 immunoprecipitation data; additional information about 3X-FLAG p52 IP.**

**A.** Representative western blots of anti-FLAG immunoprecipitated material, eluted from the resin with anti-FLAG peptide, from cells expressing the indicated FLAG-tagged p52 protein. Levels of XPB are decreased in p52 mutants (red box). These data are similar to Figure 4, which examined the material bound (but not peptide eluted) to the FLAG resin.

**B.** Quantitation of western blot data from wild-type and mutant TFIIH complexes. For quantitation, band density was normalized to p52 wild-type signal. XPB abundance was significantly reduced in mutant complexes (asterisk: padj ≤ 0.04).

**C.** Overview of the human TFIIH structure (CDK7 and CCNH not shown), from PDB 6NMI.

**D.** A zoom-in on the region from panel C colored in purple, which shows the N- and C-termini of p52. The C-terminus was helical whereas the N-terminus was disordered, and neither region represented a protein-protein interface in the complex. Nevertheless, tagging at either position disrupted CAK association, for unknown reasons.

**E.** Western blot data of FLAG bead-bound material vs. flow-through reveals that a FLAG tag on either the N-terminus or the C-terminus of p52 disrupts CAK association (CAK subunit CDK7 in flow-through fraction), whereas core TFIIH subunits are not negatively affected.

**
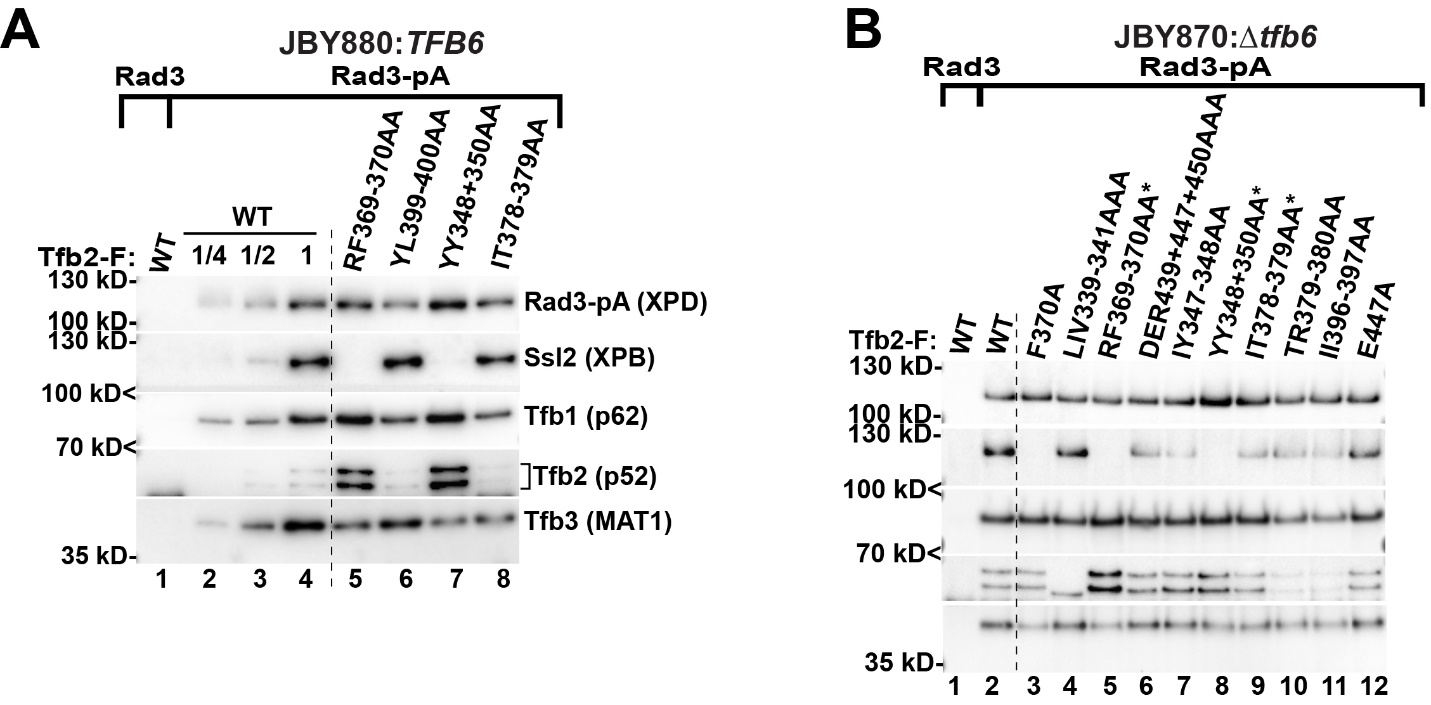
**

**Figure S6. Uncropped images corresponding to Figure 5, TFIIH integrity analysis in strains expressing Tfb2 HubA mutants.**

**A.** Whole-cell extracts from *Tfb6+* strains expressing the indicated Flag-tagged Tfb2 derivatives and pA-tagged Rad3 were subjected to IP and Western analysis using antisera against the indicated TFIIH subunits. Extracts prepared from a strain expressing untagged Rad3 and Flag-tagged WT Tfb2 were used as a control.

**B.** Whole-cell extracts from Δ*tfb6* strains expressing the indicated Flag-tagged Tfb2 derivatives and pA-tagged Rad3 were subjected to IP and Western analysis using antisera against the indicated TFIIH subunits. Extracts prepared from a strain expressing untagged Rad3 and Flag-tagged WT Tfb2 were used as a control. Asterisks indicate the mutants displayed in Figure 5C.

**
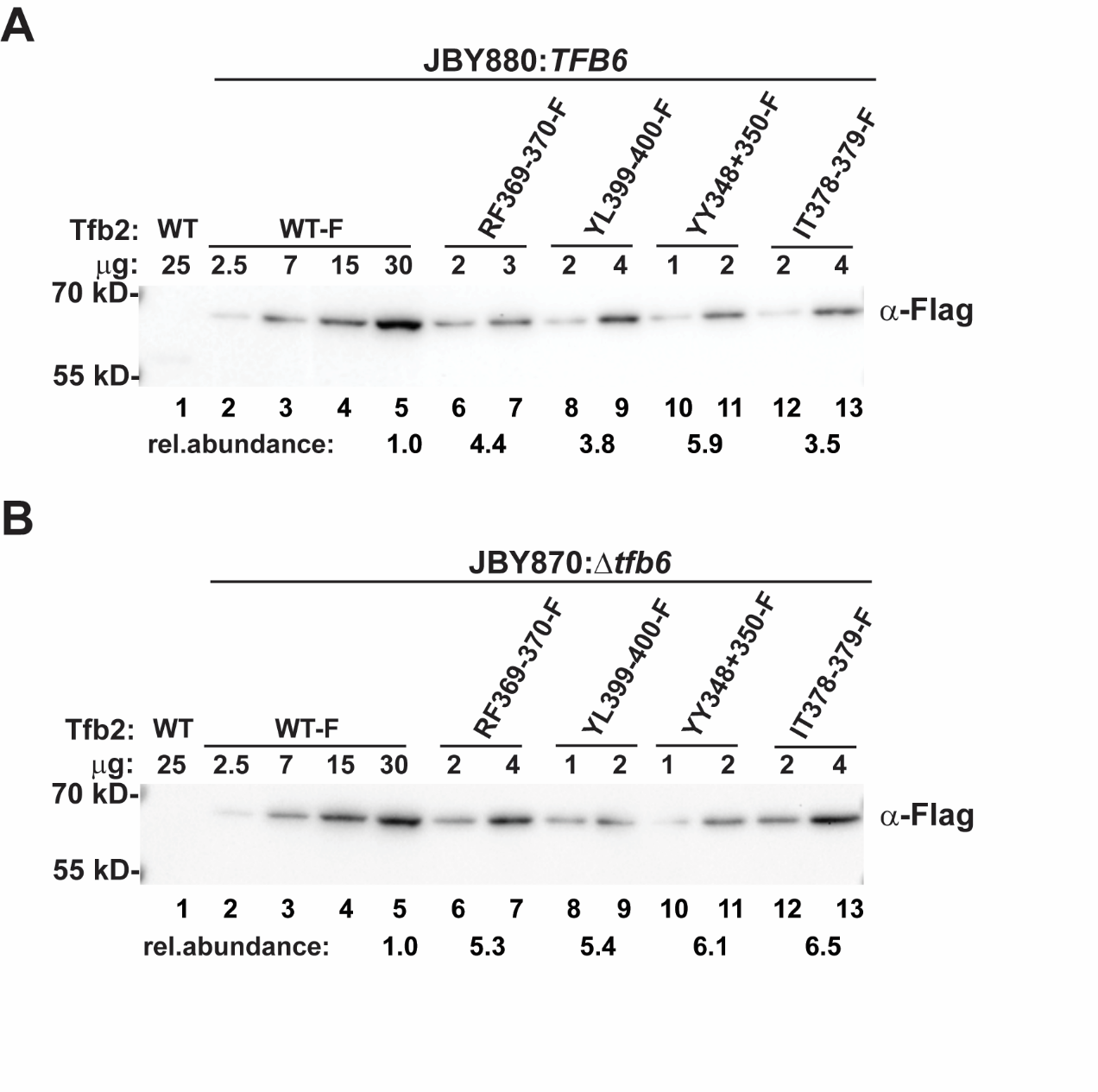
**

**Figure S7. WT and mutant Tfb2 protein levels in the presence and absence of Tfb6.**

**A-B**. Whole cell extracts prepared from JBY880 (**A**) or JBY870 (**B**) expressing FLAG-tagged WT or mutant Tfb2 were analyzed by western blotting.

**
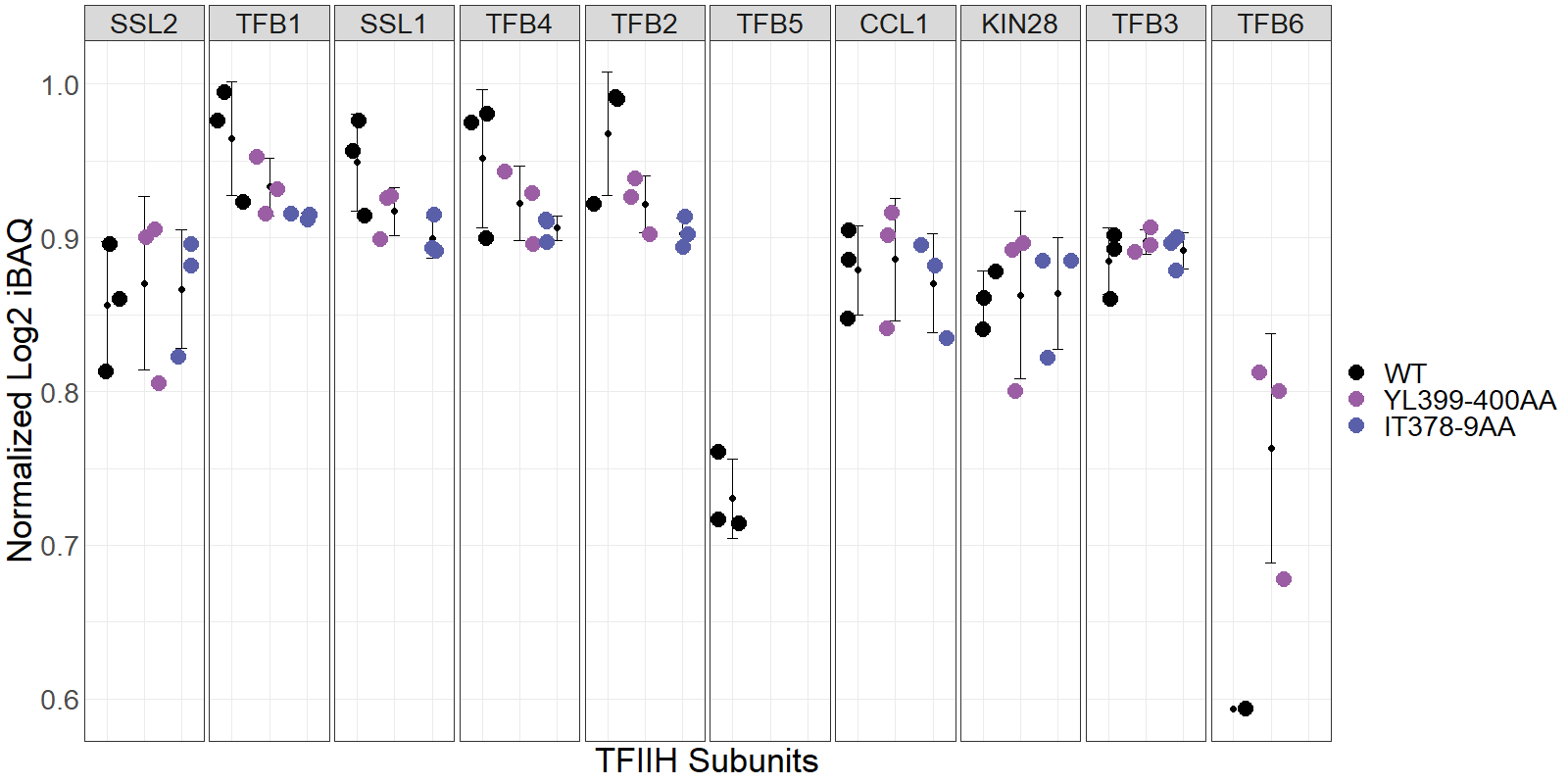
Figure S8. LC-MS results from Rad3-pA IPs from strains expressing group 2 HubA mutants.** iBAQ results (intensity Based Absolute Quantitation) normalized to Rad3 abundance, are shown comparing cells expressing WT Tfb2 to the indicated HubA mutants. The abundances of all subunits, except Tfb5 and Tfb6, are similar in each samples. Tfb5 is only detected in IPs from the WT strain, Tfb6 is reproducibly detected in IPs from the YL399-400AA mutant. n=3, biological replicates.

**
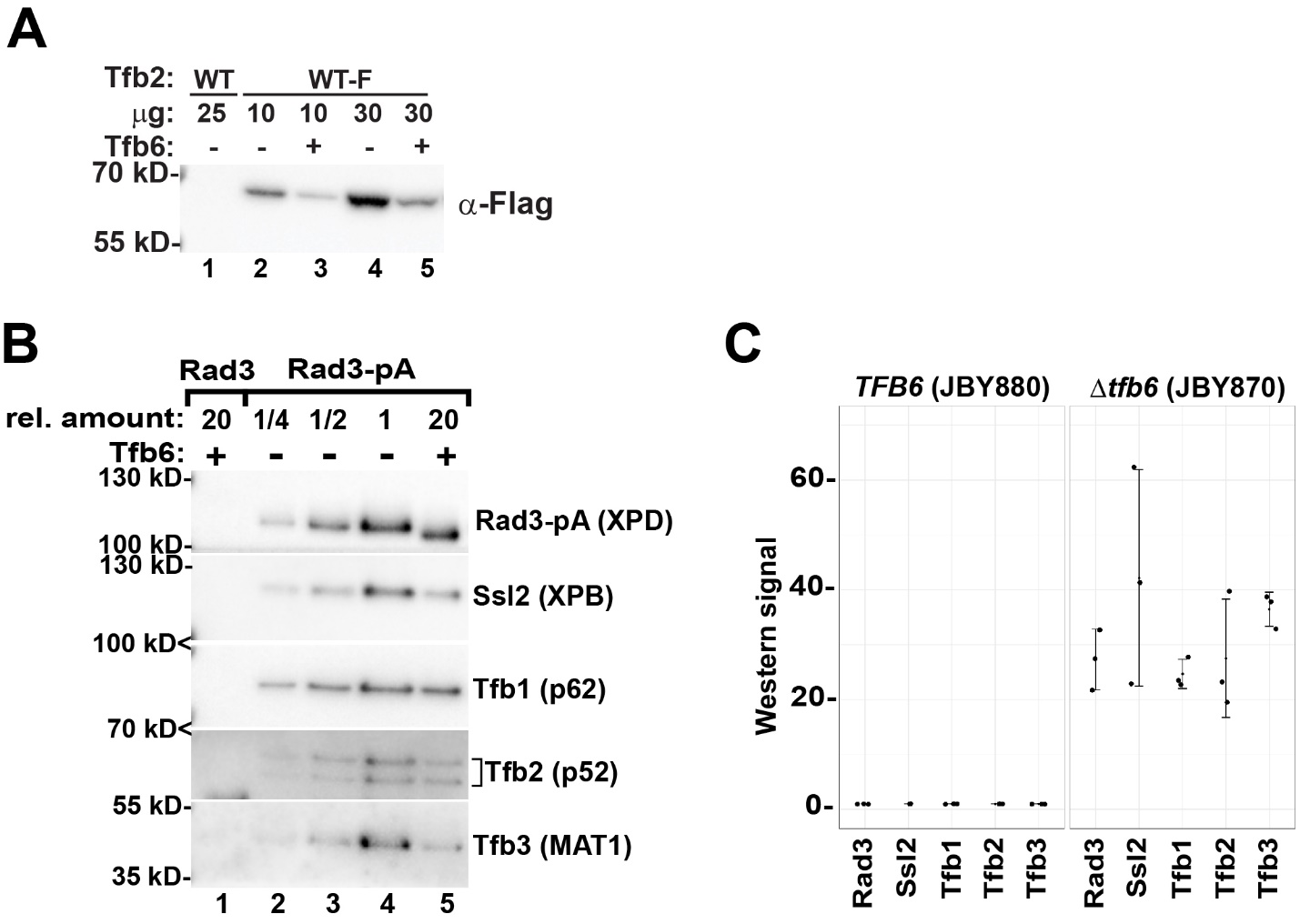
**

**Figure S9. Tfb2 abundance and Rad3-pA immunoprecipitations in the presence and absence of Tfb6.**

**A.** Whole cell extracts prepared from JBY870 (*tfb6*Δ) or JBY880 (*TFB6*) expressing FLAG-tagged WT Tfb2 were analyzed by western blotting

**B.** Whole-cell extracts from *TFB6+* and *tfb6*Δ strains expressing WT Flag-tagged Tfb2 and pA-tagged Rad3 were subjected to IP and Western analysis using antisera against the indicated TFIIH subunits. Extract prepared from a strain expressing untagged Rad3 was used as a control.

**C.** Quantitation of Western analysis.


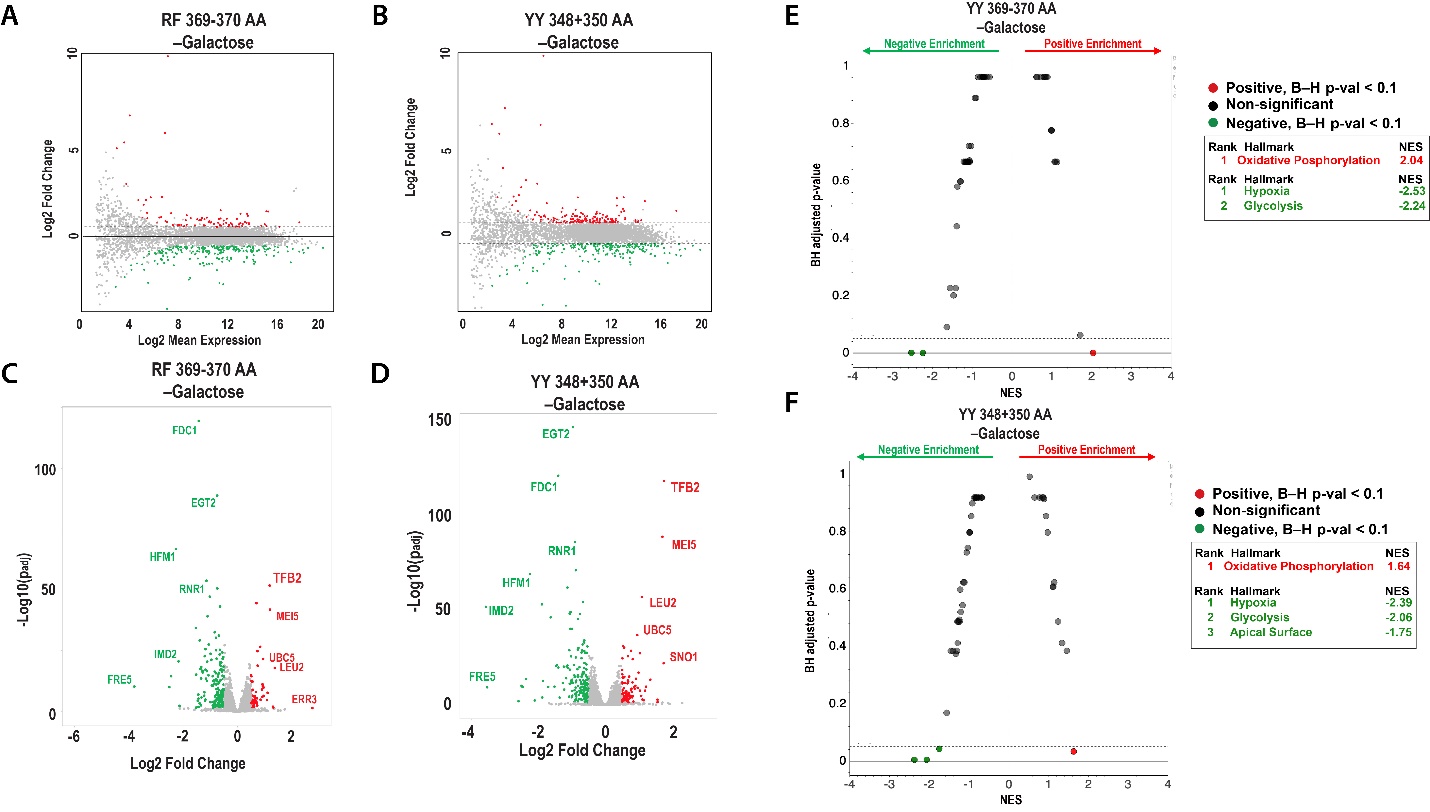


**Figure S10. RNA-seq analysis of strains expressing Tfb2 HubA mutants in the absence of galactose induction.**

**A-B.** MA plots illustrate changes in gene expression in the yeast *tfb2* HubA mutants RF369-370AA **(A)** or YY348+350AA **(B)** compared to wild-type *TFB2* in the absence of galactose treatment. Red dots signify increased gene expression and green dots represent decreased expression (padj. < 0.05 and absolute (log2foldchange) > 0.5).

**C-D.** Volcano plots show increased (red dots) and decreased (green) gene expression in *tfb2* HubA mutants in the absence of galactose treatment, compared to wild-type *TFB2* cells.

**E-F.** Gene Set Enrichment Analysis (GSEA) comparing HubA mutants RF369-370AA **(E)** and YY348+350AA **(F)** vs. wild-type *TFB2* cells. Pathways with Benjamini-Hochberg (BH) adjusted p-value <0.1 are shown as colored dots and listed in the Table (inset). NES: GSEA Normalized Enrichment Score.

**
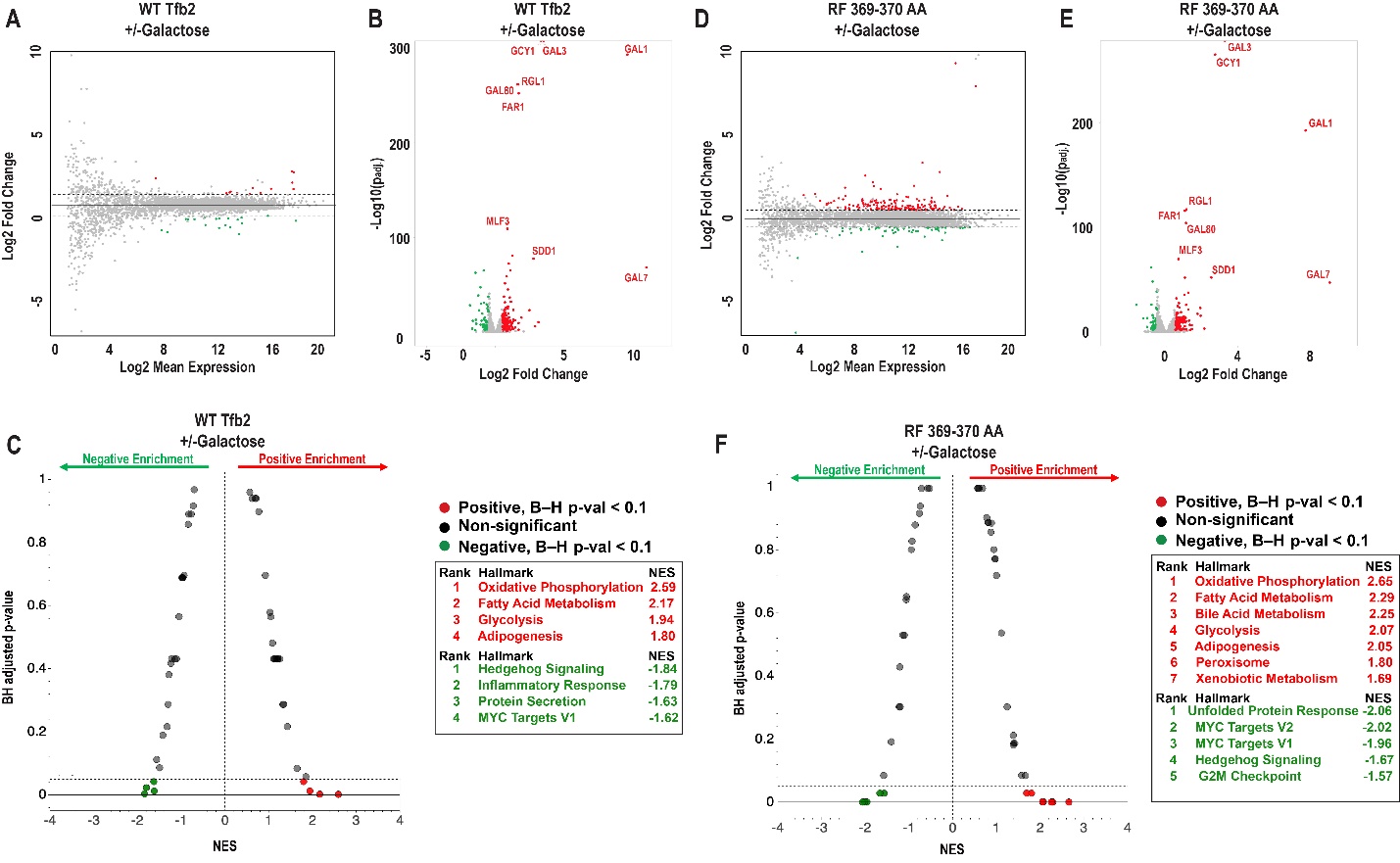
**

**Figure S11. Additional RNA-seq analysis of wild-type *TFB2* and HubA mutants ± galactose induction.**

**A.** MA plot showing gene expression changes ± 60 min galactose treatment in wild-type *TFB2* cells.

**B.** Volcano plot showing increased (red dots) and decreased (green) transcripts in wild-type *TFB2* cells, ± galactose treatment.

**C.** Gene Set Enrichment Analysis (GSEA) comparing wild-type *TFB2* cells ± galactose treatment. Pathways with Benjamini-Hochberg (BH) adjusted p-value <0.1 are shown as colored dots and listed in the Table (inset). NES: GSEA Normalized Enrichment Score.

**D.** MA plot showing gene expression changes ± 60 min galactose treatment in RF369-370AA HubA mutant cells.

**E.** Volcano plot showing increased (red dots) and decreased (green) transcripts in RF369-370AA HubA mutant cells, ± galactose treatment.

**F.** Gene Set Enrichment Analysis (GSEA) comparing *tfb2* HubA RF369-370AA mutant cells ± galactose treatment. Pathways with Benjamini-Hochberg (BH) adjusted p-value <0.1 are shown as colored dots and listed in the Table (inset). NES: GSEA Normalized Enrichment Score.

**
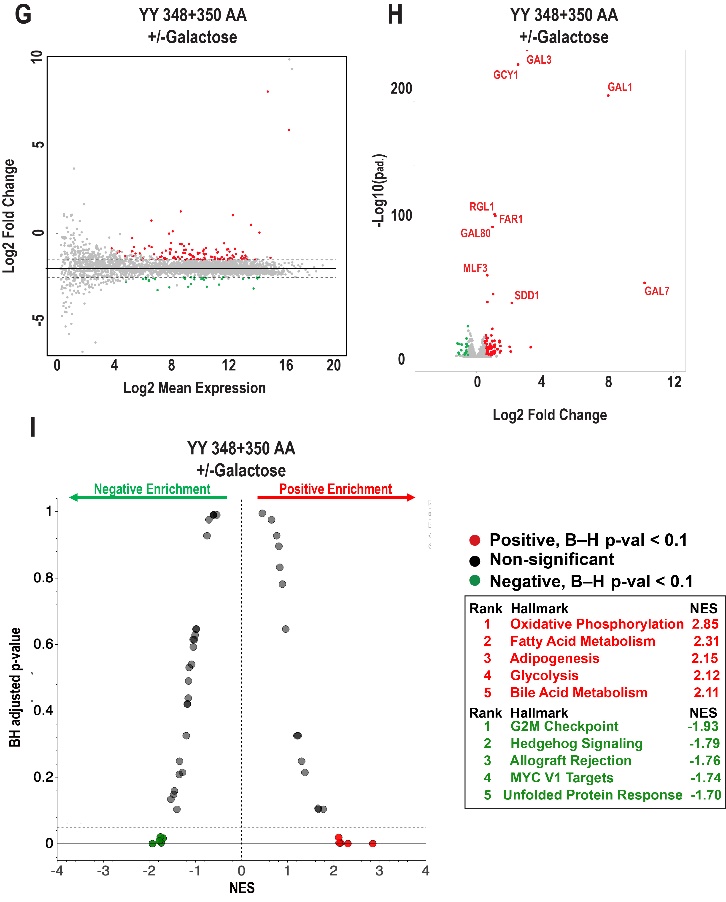
**

**Figure S11 (cont.). Additional RNA-seq analysis of wild-type *TFB2* and HubA mutants before and after galactose induction.**

**G.** MA plot showing gene expression changes ± 60 min galactose treatment in YY348+350AA HubA mutant cells.

**H.** Volcano plot showing increased (red dots) and decreased (green) transcripts in YY348+350AA HubA mutant cells, ± galactose treatment.

**I.** Gene Set Enrichment Analysis (GSEA) comparing *tfb2* HubA YY348+350AA mutant cells ± galactose treatment. Pathways with Benjamini-Hochberg (BH) adjusted p-value <0.1 are shown as colored dots and listed in the Table (inset). NES: GSEA Normalized Enrichment Score.

**
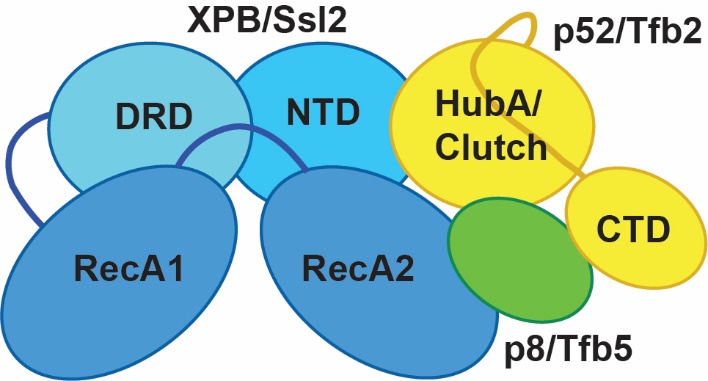
**

**Figure S12. Schematic of the XPB/Ssl2-p52/Tfb2 interaction within TFIIH.** Adapted from Greber *et. al*.(1).

**References**

1. Greber, B. J., Toso, D. B., Fang, J., and Nogales, E. (2019) The complete structure of the human TFIIH core complex. *Elife* **8**
